# SoPPIs: a highly parallelized protein–protein-interaction screening method in prokaryotic and eukaryotic hosts

**DOI:** 10.1093/nar/gkag716

**Published:** 2026-07-22

**Authors:** Silvio Collani, Sarah Muniz Nardeli, K V S K Arjun Chowdary, Daniela Goretti, Markus Schmid

**Affiliations:** Umeå Plant Science Centre, Department of Plant Physiology, Umeå University, Umeå SE-90187, Sweden; DISSTE, University of Eastern Piedmont, Vercelli 13100, Italy; Umeå Plant Science Centre, Department of Plant Physiology, Umeå University, Umeå SE-90187, Sweden; Department of Plant Biology, Linnean Center for Plant Biology, Swedish University of Agricultural Sciences, Uppsala S-75007, Sweden; Department of Plant Biology, Linnean Center for Plant Biology, Swedish University of Agricultural Sciences, Uppsala S-75007, Sweden; Umeå Plant Science Centre, Department of Plant Physiology, Umeå University, Umeå SE-90187, Sweden; DISSTE, University of Eastern Piedmont, Vercelli 13100, Italy; Umeå Plant Science Centre, Department of Plant Physiology, Umeå University, Umeå SE-90187, Sweden; Department of Plant Biology, Linnean Center for Plant Biology, Swedish University of Agricultural Sciences, Uppsala S-75007, Sweden

## Abstract

Protein–protein interactions (PPIs) are at the heart of most cellular processes but despite recent progress, their genome-wide analysis remains challenging. With this in mind, we have developed SoPPIs (sequencing of PPIs), a powerful method that facilitates parallelized PPI analyses using an innovative combination of the split-Cre/loxP system and high-throughput DNA sequencing. Sequential recombination of plasmids encodes information about pairs of interacting proteins in recombined DNA, facilitating their easy and cost-efficient identification by next-generation sequencing. Importantly, SoPPIs works with most soluble proteins, can be implemented in any cell type that can be transformed with episomal plasmids, and is in principle capable of interrogating all possible PPIs in an organism in a single experiment. To demonstrate the power of SoPPIs, we analyzed the composition of the *Arabidopsis thaliana* LSm/Sm ring, an evolutionarily highly conserved core component of the spliceosome and performed parallelized library screens to identify LSm/Sm-interacting proteins. Given its versatility and usability, we expect SoPPIs to quickly gain popularity and help provide insights into the PPI networks underlying complex biological systems.

## Graphical Abstract

**Graphical Abstract:**
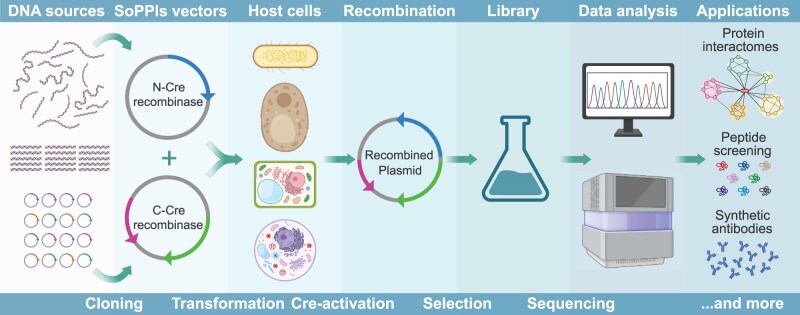

## Introduction

Technological advances over the past two decades have enabled genome-wide analyses at an unprecedented scale. High-throughput short-read sequencing, in particular, has revolutionized how we think about and analyze nucleic acids. Sequencing of complete (epi-) genomes has become state of the art empowering, the study and comparison of entire genomes across different species. Similarly, RNAseq, and more recently single-cell and single-nucleus sequencing and spatial transcriptomics, facilitates the robust and cost-effective analyses of gene expression of entire transcriptomes across various tissues and experimental conditions at unprecedented scale and temporal and spatial resolution.

In contrast to the significant advances in the analysis of nucleic acids, analyses of entire proteomes in general and protein–protein interactions (PPIs) in particular are lagging despite impressive progress. Conventional methods for investigating PPIs, such as yeast two-hybrid screens (Y2H), bacterial two-hybrid screens (B2H), split-ubiquitin and split-luciferase systems, FRET-based approaches, bimolecular fluorescence complementation, and co-immunoprecipitation coupled to mass spectrometry [1–6] offer valuable insights into the composition and function of specific proteins and protein complexes but usually lack the scalability necessary for comprehensive proteome-wide analyses. Several methods that enable the parallelization of the analyses of pairs of proteins or simultaneous analysis of interactions between multiple proteins have been developed [7–12]. However, these methods are currently not widely used as they usually require a complex experimental setup, making them costly, labor-intensive, or both. Moreover, existing methods for multiplexing PPI analyses in heterologous systems are usually limited to a specific host. In the last years methods for *in silico* PPIs prediction have also been developed, as well as *in silico* prediction to study DNA–protein interactions [13–17], but they are still lacking biological validation [18–22]. To address these limitations, we have developed SoPPIs (sequencing of protein–protein interactions), an innovative method that combines the power of the Cre/loxP recombinase system with high-throughput short-read sequencing.

Cre recombinase derived from bacteriophage P1 facilitates DNA excision or inversion *in cis* and recombination of two DNA molecules *in trans*. Its activity depends on the presence of two 34 bp loxP sequences, each comprising 13 bp inverted repeats flanking an 8 bp central spacer region. Depending on the positioning and orientation of these loxP sites, the Cre recombinase can perform various actions, including excision, inversion, or recombination of DNA fragments [23]. Mutations in the loxP sequences have led to the identification of three classes of variants: spacer mutants, left-inverted repeat mutants, and right-inverted repeat mutants, each affecting the activity of the Cre recombinase differently [24–26]. These mutant loxP variants differ strongly in their affinity to one another [27, 28]. For example, the affinity between the lox66 and lox71 variants far exceeds the affinity of either of these variants for the loxWT sequence [28], making it possible to combine different loxP variants so that recombinations within and between molecules occur in a predictable order.

Crucially, analogous to the GAL4 transcription factor used in Y2H or adenylate cyclase in B2H, Cre recombinase can be split into two inactive halves, each of which can be fused to other proteins. Upon interaction of these fusion partners, the function of the Cre recombinase is reconstituted, enabling DNA recombination [10, 29, 30]. Importantly, the propensity of the individual split-Cre fragments to complement each other in the absence of interacting fusion partners is exceedingly low, resulting in low background activity [29].

Here, we report the implementation of SoPPIs in *Escherichia coli* and *Saccharomyces cerevisiae* as examples of prokaryotic and eukaryotic hosts. Specifically, we verified SoPPIs specificity using a diverse set of proteins and constructed a comprehensive protein interaction matrix for 25 splicing-related LSm/Sm proteins from Arabidopsis (*Arabidopsis thaliana* (*L*.) *Heynh*.) (Supplementary Table S1) using the bacteria *E. coli* as the host. Key results from this interaction matrix study were subsequently validated using SoPPIs in *S. cerevisiae*. Furthermore, to demonstrate the high throughput capabilities of SoPPIs, we performed parallelized complementary DNA (cDNA) library screens in two biological replicates using the 25 Arabidopsis LSm/Sm proteins as baits, interrogating a total of ~180 million potential PPIs. Our results not only provide valuable insights into the Arabidopsis splicing machinery but also demonstrate that SoPPIs has the potential to become a routine method for generating comprehensive protein interactomes at significantly reduced time and cost.

## Materials and methods

### Bacterial SoPPIs vectors

For the design of the two bacterial SoPPIs vectors, two non-functional fragments of Cre recombinase protein (343 amino acids; AA) were used: the first vector contains the N-terminal fragment (N-Cre; AA 1–59) of the full-length Cre recombinase, followed by a lox66 site, and the coding sequence of the first target gene; the second vector contains the C-terminal fragment (C-Cre; AA 60–343) followed by a lox71 site, and the coding sequences of the second gene of interest (Supplementary Fig. S1A and B). Both, the N-terminal and C-terminal fragments were codon-optimized for efficient translation in *E. coli*.

The N-Cre vector (pSC_N-CRE_B) was built using pUC19 as a backbone, in which we replaced the LacZ cassette with a cassette encoding the N-terminal fragment of Cre-recombinase (AA 1–59) under the control of the Isopropyl β-D-1-thiogalactopyranoside (IPTG) inducible lac operon, followed by a sequence encoding a 12 AA linker, a lox66 site in 5′ to 3′ direction, and attR1 and attR2 Gateway recombination sequences flanking a ccdB/chloramphenicol cassette flanked by restriction enzyme (RE) sites suitable for cloning using digestion with REs, followed by ligation using T4 DNA ligase. Gateway LR reaction will introduce the open reading frame (ORF) of the first target genes in frame with N-Cre recombinase. The vector carries the *bla* gene under the constitutive *pBla* promoter, conferring resistance against ampicillin and carbenicillin (Supplementary Fig. S1A).

The C-Cre SoPPIs vector (pSC_C-CRE_B) is based on the plasmid pETM-11, from which we removed the expression cassette, including the 6× His tag and TEV site. Into this truncated vector, we introduced the inducible lac operon to allow the expression of target genes in bacterial strains lacking T7 polymerase and a cassette encoding for C-terminal fragment of Cre-recombinase (AA 60–343, plus an ATG starting codon), followed by a 12 AA linker, a lox71 site in 3′ to 5′ direction, and attR1 and attR2 Gateway recombination sequences flanking a ccdB/chloramphenicol cassette flanked by RE sites suitable for cloning using digestion with REs, followed by ligation using T4 DNA ligase. Gateway LR reaction will bring the ORF of the second target gene in frame with C-Cre recombinase. We also added a constitutive *pKat* promoter and ribosome binding site to the *aph(3′)-Ia* gene, conferring resistance to kanamycin. Finally, we introduced a loxWT site followed by the *ant(3′’)-Ia* gene conferring spectinomycin resistance (Supplementary Fig. S1B). The *ant(3′’)-Ia* gene was cloned without promoter and start codon to prevent its expression and translation until after sequential *in trans* and *in cis* recombinations, which place the *ant(3′’)-Ia* coding sequence in frame with the N-terminal half of Cre recombinase and under the control of the lac operon, facilitating IPTG-inducible expression.

### Yeast SoPPIs vectors

Following the design principles used in the bacterial vectors, two SoPPIs vectors were created for use in *S. cerevisiae* (Supplementary Fig. S1C and D). The N- and C-terminal fragments of Cre recombinase used in these vectors were codon-optimized for translation in *S. cerevisiae*.

To build a first SoPPIs vector suitable for work in yeast (pSC_N-CRE_Y), we replaced the GAL4-BD domain of pDEST-BD with the N-terminal fragment (AA 1–59) of Cre-recombinase, followed by a 12 AA linker, a lox66 site in 5′ to 3′ direction, and attR1 and attR2 Gateway recombination sequences flanking a ccdB/chloramphenicol cassette flanked by RE sites suitable for cloning using digestion with REs, followed by ligation using T4 DNA ligase. Gateway LR reaction will bring the coding sequence of the first target gene in frame with N-Cre. An SV40 NLS peptide signal has been added in frame at the N-terminus of the N-Cre recombinase fragment to ensure nuclear localization of the fusion protein. The vector contains the *LEU2* gene (aux-L) under the constitutive *pLEU2* promoter as a selectable marker in *S. cerevisiae* and the *bla* gene under the constitutive *pBla* promoter conferring resistance against ampicillin and carbenicillin in *E. coli* (Supplementary Fig. S1C).

The C-Cre vector for SoPPIs in yeast (pSC_C-CRE_Y) is based on pDEST-AD, which was modified as follows: the GAL4-AD domain was replaced with the C-terminal half of Cre-recombinase (AA 60–343 plus an ATG starting codon), followed by a 12 AA linker, a lox71 site in 3′ to 5′ direction and attR1 and attR2 Gateway recombination sequences flanking a ccdB/chloramphenicol cassette flanked by RE sites suitable for cloning using digestion with REs, followed by ligation using T4 DNA ligase. Gateway LR reaction will bring the coding sequence of the target gene in frame with C-Cre. An SV40 NLS peptide signal has been added in frame at the N-terminus of the C-Cre recombinase fragment. The vector contains the *TRP1* gene (aux-W) under the constitutive *pTRP* promoter as a selectable marker in *S. cerevisiae* and the *bla* gene under the constitutive *pBla* promoter conferring resistance against ampicillin and carbenicillin in *E. coli*. Between the origin of replication *CEN/ARS* and the *TRP1* gene, we added a loxWT site followed by the *Sh-ble* gene conferring zeocin resistance. The *Sh-ble* gene was cloned without promoter and starting codon to prevent its expression and translation until after sequential *in trans* and *in cis* recombinations. In case of sequential recombination, the *Sh-ble* gene will be fused in frame with the N-terminal half of the Cre recombinase under the control of the constitutive *pADH1* promoter (Supplementary Fig. S1D). Zeocin was chosen as selectable marker as it proved to be more stable and efficient for yeast selection in liquid culture than hygromycin (Supplementary Fig. S2).

### Analysis of pairwise PPIs using SoPPIs in *E. coli*

To test SoPPIs in direct pairwise PPI assays, the ORFs of three *A. thaliana* Sm proteins, SmE, SmF, and SmG were amplified from Arabidopsis cDNA and cloned into pSC_N-CRE_B and pSC_C-CRE_B vectors using restriction-ligation cloning. ORFs of 20 additional *A. thaliana* genes were ordered as synthetic constructs and cloned (GenScript Biotech, The Netherlands) into pDONR/Zeo (Thermo Fisher Scientific) and finally transferred into pSC_N-CRE_B and pSC_C-CRE_B by Gateway LR cloning.

Direct PPI analyses between pairs of proteins were performed as follows: vectors providing N-Cre and C-Cre fragments fused in frame to the coding sequences of two proteins of interest were co-transformed by heat-shock in 50 μl of *E. coli* DH5α competent cells, 950 ml of LB or SOC media was added to each co-transformation, and the starting cultures were incubated at 37°C in agitation at 200 rpm for 1 h.

The starting culture was then split into two aliquots of 500 μl used to inoculate 5 ml of LB containing carbenicillin (100 ng/μl), kanamycin (50 ng/μl), and IPTG (100 μM) to select for cells containing both plasmids and to induce protein production. Cultures were incubated at 37°C in agitation at 200 rpm for 5–7 h, before being stored at 4°C overnight. After this initial incubation, 5 μl of spectinomycin (final concentration 100 ng/μl) was added to one of the two 5 ml aliquots to select for cells containing plasmids that had undergone sequential *in trans* and *in cis* recombinations and had thus activated expression of the third selectable marker, *ant(3′’)-Ia*, which provides spectinomycin resistance. The second aliquot served as control. Cells were incubated for an additional 24–30 h (incubation time depends on the efficiency of the co-transformation) at 37°C at 200 rpm, at which point the control reached an OD_600_ of 1.5–2. The second aliquot, i.e. the culture to which spectinomycin had been added, grows more slowly as only those cells in which sequential recombination has occurred will survive spectinomycin selection. To visualize the effect of sequential *in trans* and *in cis* recombinations on cell growth in control and test aliquots, in Sm proteins panel, serial dilutions of cells were prepared using LB containing the appropriate antibiotics and spotted on LB plates containing either carbenicillin, kanamycin, or carbenicillin, kanamycin, IPTG, and spectinomycin. Plates were incubated at 37°C for 16–20 h and pictures were taken using a digital camera.

Alternatively, for PPIs reported in Supplementary Fig. S3, 750 μl of the starting culture was used to inoculate 3 ml of LB broth containing carbenicillin (100 ng/μl), kanamycin (50 ng/μl), and IPTG (100 μM), cultivated for 3–5 h at 37°C in agitation at 200 rpm, and stored overnight at 4°C. In parallel, 50 μl of the co-transformed culture was spread on an LB plate with carbenicillin and kanamycin selection to determine the co-transformation efficiency. Concentration of colony forming units was adjusted by adding LB broth and 10 μl of adjusted culture was spotted on LB plates containing carbenicillin (100 ng/μl), kanamycin (50 ng/ μl), IPTG (100 μM), and spectinomycin (150 ng/ μl). Another plate without spectinomycin served as a positive control. Plates were incubated overnight at 37°C, and pictures were taken at the same time.

### Analysis of pairwise PPIs using SoPPIs in yeast

Pairwise PPI analyses using SoPPIs in *S. cerevisiae* were performed as follows: *S. cerevisiae* AH109 cells were co-transformed by heat shock using plasmids carrying the N-Cre and the C-Cre fragments of Cre recombinase fused to the coding regions of the two proteins of interest. Following co-transformation, cells were resuspended in 1 ml of sterile water, divided into two aliquots of 500 μl that were used to inoculate 5 ml of SD media lacking the amino acids leucine (-L) and tryptophan (-W). Cells were incubated at 30°C in agitation at 150 rpm for 7–9 h. After this initial incubation, 5 μl of zeocin (final concentration 100 ng/μl) were added to one 5 ml aliquot. The second aliquot (control) was left undisturbed. Cultures were incubated for an additional 36–40 h at 30°C at 150 rpm (incubation time depends on the efficiency of the co-transformation), at which point the control reaction should have reached an OD_600_ of 1.5–2. Cultures containing zeocin should grow only if sequential recombination has occurred. Serial dilutions of control and test cultures were plated on SD plates lacking leucin and tryptophan, and SD plates lacking leucin and tryptophan supplemented with zeocin (100 ng/μl), respectively, and incubated at 30°C for 24–30 h before pictures were taken.

### *Arabidopsis thaliana* cDNA library preparation

A cDNA library was generated from messenger RNA (mRNA) derived from Arabidopsis seedlings, accession Col-0, grown at 16°C in long-day (16 h light, 8 h darkness) conditions. To obtain a more comprehensive representation of mRNAs, samples were collected at 11 and 13 days after sowing and 3, 7, and 10 h after daylight started. mRNA samples were pooled and used to create a Gateway-compatible cDNA entry library using the CloneMiner cDNA library Construction Kit (Invitrogen) according to the manufacturer’s protocol. The final cDNA library in pDEST22 vector showed a titer of 4.9 × 10^6^ cfu × ml^−1^.

### Parallelized SoPPIs in *E. coli*

Multiplexing of SoPPIs required several modifications and additions compared to the pairwise analyses described above. The protocol can be divided into five main steps.

#### Preparation of the plasmid pools

For the PPI analysis of 25 Arabidopsis splicing-related LSm and Sm proteins, the ORFs of 11 LSm and 14 Sm genes (Supplementary Table S1), were cloned into the pSC_N-CRE_B and pSC_C-CRE_B by RE digestion, followed by ligation using T4 DNA ligase. Pools of the N-Cre and C-Cre plasmids, each containing all 25 *LSm* and *Sm* genes at equimolar concentration were prepared. For parallelized cDNA library screens, the cDNA library from Arabidopsis seedlings was transferred into the pSC_N-CRE_B vector by Gateway LR reaction following the manufacturer’s instructions. The concentration of the LSm/Sm plasmid pools and the cDNA library in pSC_N-CRE_B were adjusted to ~1 μg/μl.

#### Co-transformation into *E. coli*

To analyze the interactions between all 25 LSm/Sm proteins in parallel (25 × 25 LSm/Sm matrix), 1 μl of the LSm/Sm plasmid pools in pSC_N-CRE-B and pSC_C-CRE-B from step 1 were co-transformed by heat-shock in *E. coli* DH5α competent cells. For the parallelized cDNA library screen 1 μl of the pool 25 LSm and Sm genes in pSC_C-CRE_B and 1 µl of the cDNA library in pSC_N-CRE_B were mixed and electroporated into 50 μl of *E. coli* ElectroMAX DH10B cells (Thermo Fisher). Following transformation cells were resuspended in 1 ml of LB media and incubated at 37°C for 1 h at 200 rpm. 20 μl of the co-transformed cells were used to calculate the co-transformation efficiency by preparing serial dilutions and plating in duplicate on LB plates containing carbenicillin (100 ng/μl) and kanamycin (50 ng/μl).

#### Selection phase

The remaining co-transformed cells were used to inoculate 250 ml LB media containing carbenicillin (100 ng/μl), kanamycin (50 ng/μl), and IPTG (100 μM), and incubated at 37°C at 200 rpm. To maximize the representation of co-transformed cells and minimize the over-representation of cells in which sequential *in trans* and *in cis* recombination occurred more rapidly, the total volume of 250 ml was divided into five 50 ml aliquots, each inoculated with 200 μl of co-transformed cells. After 5–7 h of incubation, depending on the co-transformation efficiency, the third antibiotic spectinomycin (final concentration 100 ng/μl) was added and the cultures were left to grow until the OD_600_ reached 0.9–1.2. Cultures were pelleted by centrifugation and plasmids were extracted using the MIDI-prep plasmid kit (EZNA) according to the manufacturer’s instructions.

#### PCR-enrichment of informative DNA sequences

In the case of the parallelized cDNA library screens, we devised an optional step to enrich for informative regions, i.e. those carrying the lox66/71 hybrid site flanked by sequences encoding interacting proteins, prior to preparation of sequencing libraries. First, we amplified the region encompassing the two ORFs and the lox66/71 hybrid site by polymerase chain reaction (PCR) using DreamTaq (Thermo Fisher) and PTO-modified oligonucleotides (forward: 5′-[PTO]GCTAGTTATTGCTCAGCGG-3′, reverse: 5′-[PTO]GGCGCGTCAGCGGGTGTTGG-3′). The reaction was performed in 50 μl volume, with an initial step at 95°C for 5 min, followed by 10 cycles at 95°C for 30 s, 55°C for 30 s, 72°C for 3 min, followed by a last step at 72°C for 10 min. Next, the PCR reaction was treated with a combination of three enzymes, by adding directly in the PCR reaction 1 μl of DpnI (Thermo Fisher), 1 μl of ExoI (Thermo Fisher), and 1 μl of LambdaExo (Thermo Fisher), incubated for 1 h at 37°C to remove the template plasmids and any linear DNA fragments not protected by PTO modifications at both ends (Supplementary Fig. S4). Finally, the remaining PCR product was purified using the Mag-Bind TotalPure kit (Omega) according to the manufacturer’s instructions. Samples were quantified using the Qubit High Sensitivity kit and 50 ng of purified DNA was used for the preparation of high-throughput DNA sequencing libraries.

#### Preparation of libraries for high-throughput DNA sequencing

High-throughput short-read sequencing libraries were prepared using a PCR-free and a PCR-based method. The PCR-free libraries were prepared directly from 1 μg plasmid DNA without enriching for informative regions (see step 4) using the TruSeq DNA PCR-free kit (Illumina^®^) according to the manufacturer’s instructions. PCR-based libraries were prepared either directly from 50 ng of sequential recombined plasmid or following enrichment for informative regions as described in step 4 using the ThruPLEX^®^ DNA-Seq Kit (Takara) according to the manufacturer’s instructions. Libraries were sequenced by Illumina 150 bp paired ends (Supplementary Table S2), and the raw reads are deposited in ENA, accession number PRJEB76943.

### Data analysis

To identify and quantify PPIs based on high-throughput sequencing data, we established a computational analysis pipeline (Supplementary Figs S5 and S6) that can be divided into four main steps: analysis of the N-Cre (step 1) and C-Cre (step 2) plasmid pools by high-throughput sequencing to identify and quantify each gene in the pools for normalization purposes; analysis of sequences obtained from sequentially recombined plasmids to identify pairs of interacting proteins (step 3); and, finally, the quantification and normalization of PPIs (step 4).

#### Analysis of the N-Cre plasmid pool

The N-Cre plasmid pools used in this study contain either the coding region of 25 LSm/Sm genes, mixed at approximately equimolar concentration, or the Arabidopsis cDNA library, in which the representation of different genes in the cDNA library is much more variable and spans several orders of magnitude, ranging from rare to extremely abundant transcripts, in the vector pSC_N-CRE-B. All N-Cre plasmids share a common part consisting of the N-Cre recombinase fragment, the lox66 site, the RE sites (in the case of the 25 LSm/Sm genes) or the remnants of the Gateway sequence remaining after LR recombination (in the case of the cDNA library) and a gene-specific part, i.e. the coding region of one of the interacting proteins. To identify and quantify each gene, the pool of 25 LSm/Sm plasmids and the cDNA library in pSC_N-CRE-B were sequenced (Supplementary Table S2 and  Supplementary Fig. S7). Raw paired-end sequencing reads were merged using PEAR [31] and three categories of sequences were created: paired-end reads that could be merged into a contig, individual forward reads, and individual reverse reads. Sequences from these three categories were combined and filtered to identify sequences that contained the lox66 site, the RE sites or Gateway sequences used for cloning, and a minimum of 20 gene-specific bases after the RE sites or Gateway sequence, referred to as informative region. The gene-specific regions following the RE site or Gateway sequence were blasted against the Arabidopsis cDNA database (Araport11, 20220914 version) using the BLASTn (nucleotide BLAST) algorithm. Blast results were used to calculate the frequency (FA) of each gene tagged with N-Cre present in either the pool of the 25 LSm/Sm genes or the Arabidopsis cDNA library.

#### Analysis of the C-Cre plasmid pool

The C-Cre plasmid pool contains the CDSs of the 25 Arabidopsis LSm/Sm proteins. These plasmids consist of a shared common part consisting of the C-Cre recombinase fragment and the lox71 site and the gene-specific CDS of LSm/Sm genes. To quantify each gene in pSC_C-CRE-B vector, paired-end sequencing reads were merged using PEAR [31] and three categories of sequences were created: paired-end sequences that could be merged into a contig, individual forward reads, and individual reverse reads. Sequences from these three categories were combined and sequences covering the lox71 site and part of the flanking LSm/Sm genes were identified and used to calculate the frequency (FB) of each LSm/Sm gene in the C-Cre plasmid pool. The filtering was performed by screening the first nine nucleotides of each ORF encoding LSm/Sm proteins, which in this case is the minimum number of nucleotides required to unambiguously identify each Arabidopsis LSm/Sm gene.

#### Analysis of the pool of sequentially recombined plasmids

SoPPIs captures the information about which proteins interacted through sequential *in trans* and *in cis* recombination and encodes this information in recombined plasmids. These plasmids contain a lox66/71 hybrid site flanked by regions from the N-Cre and the C-Cre vectors that are shared by all double-recombined plasmids. These common elements are flanked by the sequences encoding the interacting proteins. In the case of the 25 × 25 LSm/Sm matrix, these are the ORFs encoding 25 Arabidopsis LSm and Sm genes. In the case of parallelized cDNA library screens, one side is comprised of the ORF of one of the 25 Arabidopsis LSm/Sm genes whereas the other side is comprised of a cDNA originating from the cDNA library following the Gateway sequence. Importantly, only sequencing reads covering the 5′ ends of the two DNAs (LSm/Sm and cDNA), separated by the lox66/71 hybrid site and the flanking regions, are informative for identifying pairs of interacting proteins. To identify these informative sequences, raw paired-end sequencing reads were merged using PEAR [31] and three sequence categories were created: paired-end reads that could be merged into a contig and individual forward and reverse reads. These three categories were combined, and informative sequences were identified using a stepwise filtering procedure: in the case of the parallelized cDNA library screens, only sequences that contained a lox66/71 site flanked by a Gateway site plus at least 10 bp of a transcript originating from the cDNA library on one side and at least 9 bp of an LSm/Sm gene on the other side were retained. The retained sequences were divided into 25 groups based on the LSm/Sm gene contributed by the pSC_C-CRE_B plasmid and sequences following the lox66/71 hybrid site and the Gateway site were blasted (BLASTn) against the Arabidopsis cDNAs database (Araport11, version 20220914). Blast hits that were identified only in the sequential recombined plasmid pool but not in the sequenced cDNA library pSC_N-CRE-B plasmid pool (step 1, see above) were removed to minimize artifacts. The remaining sequences were counted and used to calculate a frequency observed (FO) for each Arabidopsis gene, which is defined as the ratio of the number of reads of each gene to the total number of sequencing reads (Supplementary Fig. S6). To establish the number of genes represented in our cDNA library, we merged different transcript isoforms derived from the same gene (Supplementary Figs S5 and S7A and B). We also analyzed whether the ORF of the cDNA was in frame with the N-Cre tag (Supplementary Figs S5 and S11D). In the case of analyzing the interaction matrix of 25 LSm/Sm proteins, filtering for Gateway sites and flanking transcripts from the cDNA library were omitted. Instead, sequences that contained a lox66/71 site flanked by at least 9 bp of an LSm/Sm gene on both sides were retained. After that, each pair of interacting LSm/Sm were assigned to one of the 625 (25 × 25) possible LSm/Sm PPI combinations using customized Python scripts.

#### Normalization and statistical analysis

The analysis pipeline outlined below was devised to assign a confidence score to the observed PPIs. To this end, we first normalized the occurrence of a given gene in the sequentially recombined sequences to the frequency of this gene in the N-Cre (FA; see step 1) and C-Cre (FB; see step 2) DNA pools. The frequency indexes FA and FB are calculated as the ratio of the number of reads of a given gene divided by the total number of reads (Supplementary Fig. S6E). The frequency expected (FE) for each possible combination of genes after sequential recombination is defined as the product obtained by multiplying the FA and FB values. FE represents the expected frequency of PPIs assuming that all proteins interact randomly, meaning the probability to observe an interaction is determined solely by abundance of the genes in the plasmid pools (Supplementary Fig. S6A, B, and E). In contrast, the FO (see step 3) reflects the actual frequency of a specific PPI detected in the experiment (Supplementary Fig. S6A, B, and E). The expected (FE) and observed (FO) frequency were used to calculate a frequency score (FS) for each PPI, which is defined as the ratio between the FO and the FE, i.e. FS = FO/FE. Thus, FS represents the normalized value for each possible PPI combination, accounting for the relative representation of each gene in the N-Cre and C-Cre DNA pools. To facilitate comparison between SoPPIs experiments that differ in complexity and sequencing depth the FS score was further normalized to values between 0 and 1, resulting in the FS_0–1_, where values close to 0 and 1 indicate low and strong support for a given PPI, respectively (Supplementary Fig. S6C).

The distribution of the FS_0–1_ values can be plotted as multiples of the median of FS_0–1_ values (Me_FS_), resulting in binning of the FS_0–1_ values with half of the FS_0–1_ values falling between 0 and 1 Me and the rest of the values are ranging from 1Me to nMe. This curve of Me_FS_ values can be represented as a series of linear equations in the form of *y* = *m*x + q, where *m* (angular coefficient; slope) corresponds to the difference between the values of Me_(n)_ and Me_(n−1)_ (Supplementary Fig. S6D). This information was used to calculate the median score (MS), which is defined as the ratio of FS_0–1_ to Me_FS_ value (Supplementary Fig. S6D and E). All data analysis results are reported in Supplementary Dataset S1.

For the downstream analyses presented here, we applied an arbitrary cut-off value of MS ≥1, i.e. only considered PPIs with FS_0–1_ values as large or larger than the median of all FS_0–1_ values as true. The Python scripts used for the data analysis presented here are available as Jupyter Notebook files [32] through FigShare, accession number: doi.org/10.6084/m9.figshare.29236658.

## Results

### SoPPIs design principles and implementation

**Figure 1.**
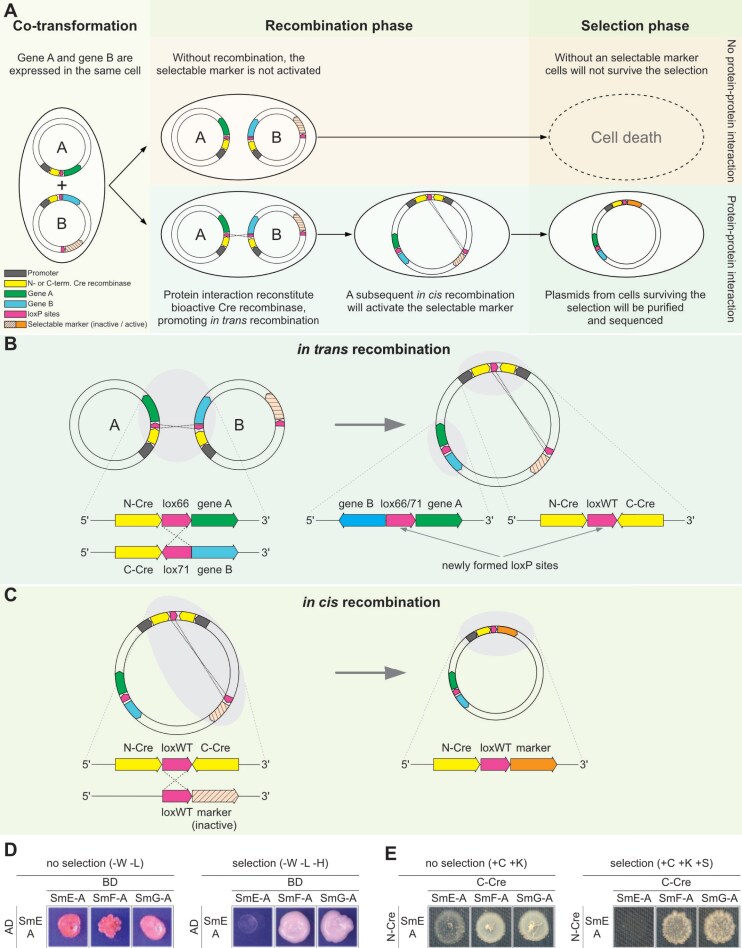
SoPPIs principle and implementation. (**A**) The three main steps of SoPPIs: co-transformation, recombination phase, and selection phase. During the co-transformation step, plasmids containing the N-terminal and C-terminal fragment of Cre recombinase (yellow) fused to ORFs encoding two test proteins (green; blue) are co-transformed into the same cells, turning each cell into a reaction vessel to interrogate one particular PPI, thereby parallelizing millions of possible combinations. During the recombination step, only interacting proteins will reconstitute active Cre recombinase promoting the sequential *in trans* and *in cis* recombination between specific loxP sites (pink), which will capture the ORFs encoding interacting proteins in a recombined plasmid and activate a selectable marker (orange). During the selection step, only those cells with an active selectable marker will survive and their sequential recombined plasmids will produce the final plasmids pool to be analyzed by high-throughput sequencing. (**B**) Details of the *in trans* recombination. Lox66 and lox71 sites (pink) oriented in opposite directions, allow the *in trans* recombination and the fusion of the two vectors. This recombination creates an intermediary vector with one new hybrid lox66/71 site (pink), flanked by the ORFs coding for the interacting proteins (green; blue) in opposite directions, and one new loxP wild type site (pink), flanked by the N-Cre and C-Cre fragments (yellow) in opposite directions. (**C**) Details of the *in cis* recombination. The two wild type loxP sites (pink) present in the intermediary vector generated in panel (B) and oriented in the same directions will promote the *in cis* recombination, which promotes the excision of a portion of the vector and brings the selectable marker (orange) in frame with the N-Cre fragment (yellow) and under the control of the inducible lac operon. In this configuration, the selectable marker becomes active, allowing the selection of those cells carrying sequentially recombined plasmids. (**D**) One-to-one PPI assays using standard Y2H system based on GAL4 activation in baker’s yeast *S. cerevisiae* using Arabidopsis SmE-A, SmF-A, and SmG-A proteins as bait/prey. Cells co-transformed with Y2H AD- and BD-vectors were grown on plates without (-W -L) or with (-W -L -H) selection for PPIs. AD: GAL4 activation domain, BD: GAL4 binding domain, W: tryptophan, L: leucin, H: histidine. (**E**) One-to-one PPI assays using bacterial SoPPIs vectors using SmE-A, SmF-A, and SmG-A proteins fused to N-Cre and C-Cre. Co-transformation following recombination and selection phases were grown on plates lacking (+C +K) or providing (+C +K +S) the activatable selectable marker (+S). C: carbenicillin, K: kanamycin, S: spectinomycin.

SoPPIs was designed to establish an easy-to-use and cost-efficient PPI assay that could be implemented in both prokaryotic and eukaryotic cells without the need for genome-encode selectable marker genes. The final design of SoPPIs includes two plasmids, in which plasmid A expresses a translational fusion between the N-terminal fragment of Cre recombinase (N-Cre) and a first test protein, separated by a lox66 site (Fig. 1A and B). Plasmid B translationally fuses the C-terminal fragment of Cre recombinase (C-Cre) to a second test protein, separated by a lox71 site. In addition, the plasmid B contains a wild-type lox (loxWT) site, followed by the coding sequence of a selectable marker that lacks a promoter and a start codon and is thus not expressed (Fig. 1A and B). Upon co-transformation, in case the two test proteins interact, Cre function is restored, promoting in a first step the *in trans* recombination between the lox66 and lox71 sites, which is strongly favored over recombination of either of these two sites with the loxWT site present on plasmid B. The resulting intermediary plasmid harbors the ORFs encoding the two test proteins in head-to-head conformation, separated by a newly formed lox66/71 hybrid site (Fig. 1B). In addition, *in trans* recombination creates a new loxWT site between the N-Cre and C-Cre fragments (Fig. 1B). In a second step, *in cis* recombination between this newly formed loxWT site and the loxWT site originating from plasmid B results in the translational fusion between N-Cre and the selectable marker, enabling positive selection of cells harboring plasmids generated by sequential *in trans* and *in cis* recombinations (Fig. 1C).

To verify the soundness of our design, we tested SoPPIs using three Arabidopsis Sm proteins, SmE-A, SmF-A, and SmG-A. Sm proteins and related LSm proteins form evolutionarily conserved heptameric ring-like protein complexes that incorporate U-rich small nuclear RNAs to form small nuclear ribonucleoprotein (snRNP) complexes that carry out important functions in mRNA maturation and degradation [33–35]. These proteins were deliberately chosen to test SoPPIs as the pairwise interactions of all seven subunits are well characterized, showing next to no variation among subunits in animals and yeast, and, based on available PPI data [35–41], are also conserved in plants. Standard Y2H confirmed that Arabidopsis SmE-A, SmF-A, and SmG-A indeed interacted as expected, i.e. SmE-A interacted with both SmF-A and SmG-A, but did not form a homodimer (Fig. 1D). Importantly, we obtained the same result using SoPPIs in *E. coli*. (Fig. 1E and Supplementary Fig. S8A and D) demonstrating that the SoPPIs principle is sound and can be used to efficiently detect PPIs in *E. coli*.

To further validate SoPPIs, we selected 20 diverse *A. thaliana* proteins organized in five quartets, for which interactions had previously been reported. Most of these interactions were originally identified using Y2H and, in many cases, were confirmed by independent methods. The selected proteins represent various biological pathways and diverse cellular localizations, including the nucleus, cytosol, and membrane, representing 80 PPIs in total (Supplementary Fig. S3).

The first two quartets consist of the four MADS domain transcription factors APETALA1 (AP1; AT1G69120.1), SUPPRESSOR OF OVEREXPRESSION OF CO 1 (SOC1; AT2G45660.1), SEPALLATA 1 (SEP1; AT5G15800.1), and SEP3 (AT1G24260.2), and HEAT SHOCK PROTEIN 90–1 (HSP90-1; AT5G52640.1), RAR1 (AT5G51700.1), SGT1A (AT4G23570.1), and SGT1B (AT4G11260.1), respectively. Importantly, for these two quartets, the results obtained using SoPPIs perfectly matched previously reported findings [42–50] (Supplementary Fig. S3A and B), demonstrating the versatility of SoPPIs. In the third quartet, consisting of the ZIM domain-containing transcriptional repressors JASMONATE-ZIM DOMAIN 1 (JAZ1; AT1G19180.1) and JAZ3 (AT3G17860.1), the adapter protein NINJA (AT4G28910.1), and the bHLH transcription factor MYC3 (AT5G46760.1), we observed interactions between the JAZ proteins and both NINJA and MYC3, as expected. However, using SoPPIs in *E. coli* we were unable to detect the formation of JAZ1 and JAZ3 homo- and heterodimers, which have been reported using Y2H [51–55], suggesting that these interactions require eukaryotic-specific post-translational protein modifications or bridging proteins [51–55] (Supplementary Fig. S3C). Similarly, analysis of PPIs between the bZIP transcription factor FD (AT4G35900.1), the 14–3-3 adapter protein GENERAL REGULATORY FACTOR 7 (GRF7; AT3G02520.2) and the two phosphatidylethanolamine-binding protein-like proteins FLOWERING LOCUS T (FT; AT1G65480.1) and TERMINAL FLOWER1 (TFL1; AT5G03840.1) confirmed homodimerization of GRF7 and interactions with the three other proteins in the quartet, while the lack of direct interaction between FD and FT/TFL1 supports the hypothesis that these interactions are mediated by eukaryote-specific 14–3-3 proteins such as GRF7 [56–59] (Supplementary Fig. S3D). Finally, to push SoPPIs to its limits, we investigated interactions between three transmembrane receptor proteins, BRASSINOSTEROID INSENSITIVE 1 (BRI1; AT4G39400.1), BRI1-ASSOCIATED RECEPTOR KINASE (BAK1; AT4G33430.1), and FLAGELLIN-SENSITIVE 2 (FLS2; AT5G46330.1), and a soluble interaction partner, BOTRYTIS-INDUCED KINASE1 (BIK1; AT2G39660.1). In agreement with results from Y2H, SoPPIs was unable to detect interactions between the trans-membrane proteins BRI1, BAK1, and FLS2, even though interactions for some of these proteins have been reported using other assays [60–65]. We did, however, detect weak homodimerization of the cytosolic protein BIK1 in both biological replicates. To our knowledge, this interaction has not been reported previously and will require further validation (Supplementary Fig. S3E).

In summary, our results demonstrate that SoPPIs in *E. coli* can be used to detect PPIs among a wide variety of soluble proteins but, as expected, cannot detect PPIs among transmembrane proteins and PPIs that require eukaryote-specific cofactors (such as GRF7) or post-translational modifications. For such assays, SoPPIs in a eukaryotic host such as yeast may be better suited (see below).

### Parallelization of PPI assays using SoPPIs

**Figure 2.**
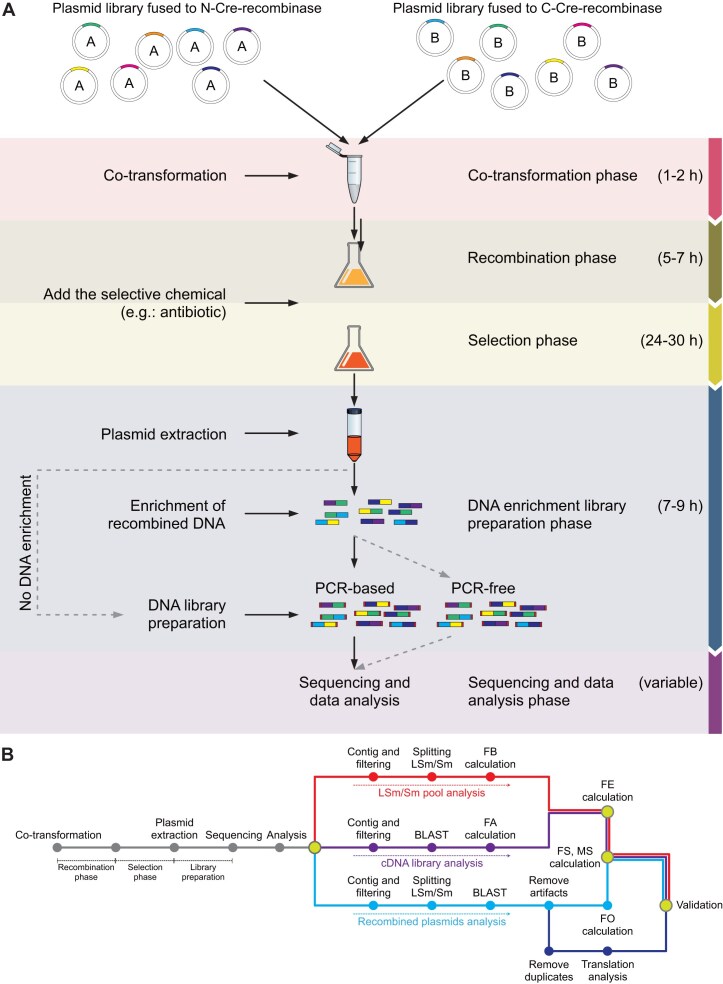
Workflow of the SoPPIs protocol and data analysis. (**A**) Graphical overview of SoPPIs protocol including options for enrichment for informative DNA fragments and high-throughput short-read sequencing library production. Dotted grey lines indicate the alternative steps used to optimize SoPPIs as described in the main text. Approximate time required for each step is provided in brackets. The duration of data analysis may vary depending on the volume of data and the processing power of the system. (**B**) Graphical overview of data analysis after high-throughput short-read sequencing. Additional details are available in the “Materials and methods” section and Supplementary Figs S5 and  S6.

An advantage of SoPPIs over many existing methods is that it can be performed entirely in liquid culture, enabling the parallelization of PPI assays without plating and picking colonies. As a proof of concept for PPI assay parallelization using SoPPIs, we performed a full matrix interaction screen using 25 Arabidopsis LSm and Sm proteins. The ORFs of 11 *LSm* and 14 *Sm* genes were cloned in frame with the N-terminal or C-terminal fragments of the Cre recombinase using plasmids A (pSC_N-CRE_B) and B (pSC_C-CRE_B) (Fig. 2A). To investigate 625 (25 × 25) PPIs in parallel, the final plasmids were co-transformed into *E. coli* (Fig. 2A, transformation phase), cultivated in liquid medium (Fig. 2A, recombination phase), before spectinomycin was added as a third antibiotic to select for those cells that had undergone sequential *in trans* and *in cis* recombinations (Fig. 2A, selection phase) as a result of PPIs between LSm/Sm proteins. Plasmid DNA was extracted in bulk and pairs of interacting LSm/Sm proteins were identified by high-throughput short-read sequencing. Sequencing libraries were prepared from 1 µg of plasmid using a PCR-free method to avoid potential biases that PCR-based methods might introduce. However, since isolating microgram amounts of plasmid DNA from host cells other than *E. coli* can be challenging, we in parallel generated high-throughput short-read sequencing libraries using a PCR-based method starting from as little as 50 ng total DNA (Fig. 2A).

We also established a bioinformatics pipeline for streamlined data analysis (Fig. 2B and Supplementary Figs S5 and S6). This analysis pipeline not only identifies pairs of interacting proteins but assigns each interaction a normalized frequency score (FS_0–1_) and a median-normalized score (MS) (Supplementary Fig. S6). FS_0–1_ represents normalized PPI FS and enables comparison between SoPPIs experiments that differ in complexity and sequencing depth, with FS_0–1_ values close to 0 and 1 indicate low and strong support for a given PPI, respectively. In contrast, MS measures the significance of a given interaction, with higher MS values indicating a stronger support for a given PPI (see the “Materials and methods” section for details).

Importantly, results obtained from the PCR-free and PCR-based sequencing library preparation methods were highly comparable and FS_0–1_ values of all possible 625 pairwise interactions of the 25 Arabidopsis LSm/Sm proteins did not exhibit substantial differences (Supplementary Fig. S9A and B and Supplementary Dataset S2). The average difference of FS_0–1_ values between the two methods was neglectable (Supplementary Fig. S9C and D), indicating that PCR amplification did not introduce any biases and can be applied for high-throughput short-read sequencing library preparation in cases where input material, i.e. sequentially recombined plasmid DNA, is limiting.

### Parallelized SoPPIs confirms conservation of snRNP assembly in plants

**Figure 3.**
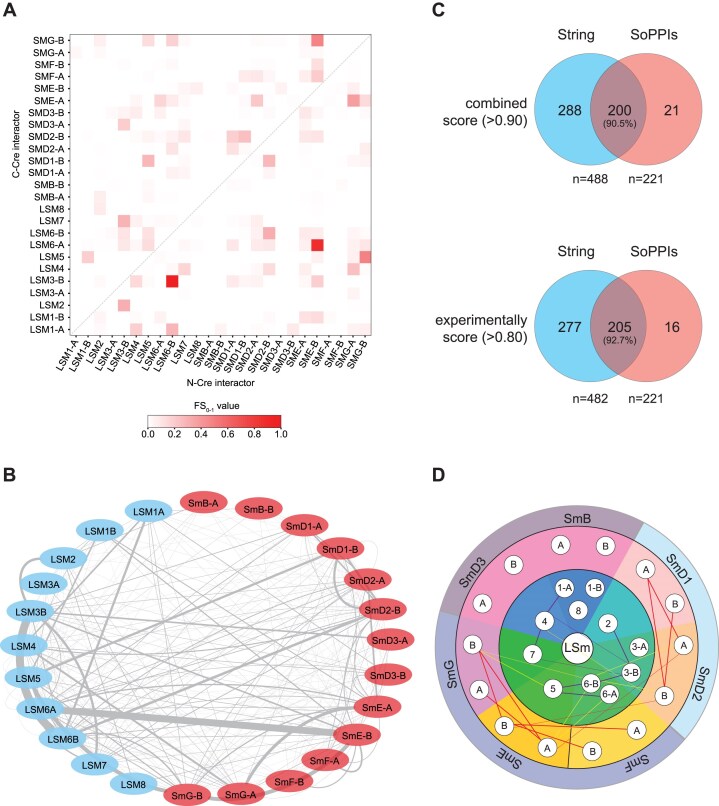
PPIs of 25 × 25 Arabidopsis LSm/Sm proteins analyzed using parallelized SoPPIs. (**A**) Matrix of PPIs of 25 × 25 Arabidopsis LSm and Sm proteins detected using parallelized SoPPIs in *E. coli* and PCR-based high-throughput short-read sequencing library preparation. The FS score indicates the confidence of the interaction, with values close to 0 indicating very unlikely (white) and values close to 1 highly likely (red) interactions. (**B**) Protein interactome among LSm (blue) and Sm (red) proteins based on 243 interactions with an MS ≥1 (Supplementary Dataset S2). Thickness of the lines is proportional to the MS value. (**C**) Venn diagrams showing overlap between 221 PPIs identified from SoPPIs with MS ≥1 (red) and PPIs listed in the String database (blue) filtered for a combined String score >0.90 (top) and experimentally String score >0.80 (bottom). (**D**) Twenty-five PPIs with the highest MS scores detected irrespectively of the orientation of the Cre-fusion partner, i.e. when the N- and C-terminal halves of the split protein are swapped, plotted onto a graphic representation of the Sm (outer) and LSm (inner) rings. Red lines: interactions among adjacent Sm proteins. Purple lines: interactions among adjacent LSm proteins. Yellow lines: interactions among proteins that occupy adjacent positions in the Sm and LSm rings. Dotted lines: proteins interactions among non-adjacent members of the protein rings.

Considering only the PPIs from PCR-based high-throughput short-read sequencing libraries (Fig. 3A) with an MS value ≥1 we identified 243 interactions between Arabidopsis LSm/Sm proteins (Fig. 3B). Over 90% of these PPIs overlapped with LSm/Sm interactions in the public String database (https://www.string-db.org) [36, 37], irrespectively of whether we used a String combined score ≥0.9, which combines the probabilities from different lines of evidence supporting a particular PPI into a single (combined) value, or a String experimental score ≥0.8, which is derive solely from experimental data (Fig. 3C and Supplementary Fig. S9E and Supplementary Dataset S2). At the same time, SoPPIs detected very few PPIs not listed in the String database (Fig. 3C and Supplementary Fig. S9E and Supplementary Dataset S2), suggesting a low false-positive detection rate. The actual false-positive rate may be even lower if some of the detected PPIs are plant-specific and have not yet been reported in the String database.

Even though the Sm/LSm proteins are evolutionarily highly conserved, an overlap of over 90% is remarkable given that the LSm/Sm interaction data in the String database is primarily based on predicted and experimentally confirmed PPIs from species other than Arabidopsis. Importantly, SoPPIs was capable of detecting in a single experiment the vast majority of interactions of Sm/LSm proteins identified by the IntAct (https://www.ebi.ac.uk/intact/home) [38] and the “Arabidopsis Interactive Mapping Consortium” [39] initiatives, showing 84.6% and 83.3% of overlap, respectively (Supplementary Fig. S9F and G), and several interactions between combinations of Sm/LSm proteins that have previously been reported. For example, using SoPPIs we detected interactions between LSM1 and LSM7 as well as LSM2 and LSM8, but not between LSM1 and LSM8, which is in line with these proteins forming distinct heteroheptameric LSM1-7 and LSM2-8 complexes that participate in mRNA quality control in the cytoplasm and pre-mRNA splicing in the nucleus, respectively. We furthermore detected—using both PCR-free and PCR-based SoPPIs—interactions of LSM7 with LSM5, SmE-A and SmE-B, as previously shown using standard Y2H [35, 40, 41].

Detection of PPIs using the reconstitution of a split protein, such as GAL4 in the case of Y2H or in the case of SoPPIs, Cre recombinase, can depend on the combination of tags and test proteins. It is therefore generally assumed that interactions detected in both orientations (i.e. when the N- and C-terminal halves of the split protein are swapped) are more reliable. Applying such a more stringent filter, we identified 67 PPIs that were independent of the orientations of the split-Cre with an MS value ≥1. Among the 25 most significant PPIs out of these 67 interactions, >75% were observed between subunits that occupy adjacent positions in the Sm and LSm rings (Fig. 3D), indicating that adjacent members of the rings are more likely to interact. In particular, we observed pairwise interactions between SmD1-SmD2 and SmF-SmE-SmG (Fig. 3D), which in metazoans form a dimer and trimer respectively [66, 67]. Thus, parallelized SoPPIs allowed us to reconstruct the PPIs of two of the three subunits that form the Sm ring in plants in a single experiment.

### SoPPIs in *S. cerevisiae* provides insights into plant Sm ring assembly

**Figure 4.**
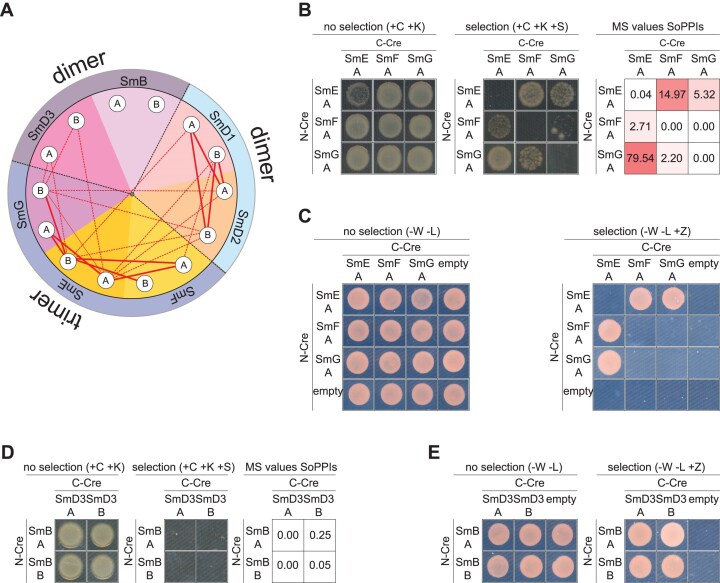
Comparison of SoPPIs in prokaryotic and eukaryotic hosts. (**A**) PPIs among Sm proteins with MS ≥1 detected irrespectively of the orientation of the Cre-fusion partner, i.e. when the N- and C-terminal halves of the split protein are swapped (*n* = 23) using parallelized SoPPIs in *E. coli* and PCR-based high-throughput short-read sequencing library preparation. Red solid lines: interactions among Sm proteins belonging to the dimers or trimer. Red dotted lines: proteins interactions outside the dimers and trimer. (**B**) Direct PPI assays for SmE-A, SmF-A, and SmG-A in *E. coli*. Left: cells grown without the selectable antibiotic, middle: after the addition of the selectable antibiotic, right: corresponding MS values extracted from the high-throughput short-read sequencing data analysis of 25 × 25 Arabidopsis LSm and Sm proteins. (**C**) Direct PPIs for SmE-A, SmF-A, and SmG-A using SoPPIs vectors optimized for use in *S. cerevisiae*. Left: cells grown without the selectable antibiotic; right: cells grown after addition of the selectable antibiotic, zeocin. (**D**) PPIs between SmB-A, SmB-B, SmD3-A, and SmD3-B assayed using SoPPIs in *E. coli*. Left: cells grown without the selectable antibiotic; middle: after the addition of the selectable antibiotic (+S); right: corresponding MS values extracted from the high-throughput short-read sequencing data analysis of 25 × 25 Arabidopsis LSm and Sm proteins. (**E**) PPIs between SmB-A, SmB-B, SmD3-A, and SmD3-B dimer assayed using SoPPIs in *S. cerevisae*. Left: cells grown without the selectable antibiotic; right: cells grown after the addition of the selectable antibiotic, zeocin. +C: carbenicillin, +K: kanamycin, +S: spectinomycin, -W: tryptophan, -L: leucin, +Z: zeocin.

Taking a closer look at the precursor complexes from which the heteroheptameric Sm-ring is assembled, we realized that SoPPIs in *E. coli* did not detect interactions between SmB-SmD3, which form the second dimer necessary for Sm ring is assembly (Fig. 4A).

To confirm the reliability of SoPPIs, we first verified all possible pairwise combinations between SmF-A, SmE-A, and SmG-A, and compared the results with the MS scores calculated based on the results from our parallelized SoPPIs analysis of 25 LSm/Sm proteins. We observed strong interaction between SmE-A and SmF-A, and SmE-A and SmG-A, but only weak interaction between SmF-A and SmG-A, and no homodimers for any of the three proteins (Fig. 4B and Supplementary Fig. S8B). Notably, these results are reflected in the MS values from our parallelized SoPPIs analysis of 25 LSm/Sm proteins (Fig. 3A), indicating that SoPPIs yields highly reproducible results and that MS values might reflect the likelihood of a given interaction. In contrast, we did not observe interactions between SmB and SmD3 in direct pairwise interaction assays using SoPPIs in *E. coli* (Fig. 4D and Supplementary Fig. S8C), confirming the results obtained from parallelized SoPPIs screens (Fig. 3D).

One possible explanation why SoPPIs in *E. coli* failed to detect interaction between SmB and SmD3 is that the paralogs of these proteins in metazoans must be methylated before the dimer is joined with the intermediary SmD1-SmD2-SmF-SmE-SmG pentamer to form the final heptameric Sm ring [68–71]. However, the protein methylase responsible for this modification is absent in *E. coli*, suggesting that lack of methylation may prevent SmB and SmD3 dimerization in *E. coli*.

To test this hypothesis, we took advantage of the fact that SoPPIs, unlike existing PPI assays, can, in principle, be adapted to any cell type that can be transformed with episomal plasmids. SoPPIs vectors were prepared for *S. cerevisiae* (baker’s yeast) and used to test all pairwise combinations of SmF-A, SmE-A, and SmG-A in this eukaryotic host (Fig. 4C). Our results not only demonstrate that SoPPIs can be implemented in eukaryotic hosts, such as baker’s yeast, but also confirmed the observations previously made in *E. coli* regarding the PPI of SmF, SmE, and SmG (Fig. 4B and Supplementary Fig. S8E). Next, we expressed SmB and SmD3 in *S. cerevisiae*, which encodes a clear ortholog of the protein methylase. Interestingly, we observed a strong interaction between SmD3 and SmB using SoPPIs in baker’s yeast (Fig. 4E and Supplementary Fig. S8F), suggesting that protein methylation is indeed required for SmD3-SmB dimerization.

These findings not only provide valuable insights into Sm ring assembly in plants, in particular the role of protein methylation, which has not yet been studied in details in plants [72–75], but also underscore the versatility and power of SoPPIs for analyzing requirements for post-translational protein modifications or the need for chaperone involvement in PPIs by comparing results obtained from prokaryotic and eukaryotic hosts.

### A LSm/Sm PPI network derived from parallelized cDNA library screens

Having established SoPPIs in *E. coli* and *S. cerevisiae* and having shown that it can be used to multiplex PPI analyses, we next applied SoPPIs to parallelize the screening of an Arabidopsis cDNAs library tagged with N-Cre using the 25 Arabidopsis LSm/Sm proteins tagged with C-Cre as baits. Sequencing of the cDNA library identified a total of 184 684 different transcripts corresponding to 15 175 distinct genes (Supplementary Fig. S7A,B), confirming its complexity. Sequencing of the LSm/Sm plasmid pool confirmed roughly equimolar representation of the 25 plasmids, fluctuating around 4% (Supplementary Fig. S7C). Consequently, we expected to screen ~400 000 PPIs (15 175 × 25), representing over 4.6 million distinct transcript–protein combinations (184 684 × 25). The frequency of individual genes in the cDNA library and the LSm/Sm plasmid pool were used to calculate the FE value, which was used for data normalization and statistical analyses (Fig. 2B and Supplementary Figs S5 and S6).

Parallelized cDNA library screens were performed in *E. coli* in duplicate. In total, ~85 and 96 million LSm/Sm–cDNA combinations were tested in the two replicates, respectively. The use of SoPPIs in parallelized cDNA library screens required further optimization of high-throughput library preparation, as most reads produced by directly sequencing the pool of plasmids generated by SoPPIs do not cover the lox66/71 site and the adjacent genes, rendering them uninformative. To increase the number of informative reads, we devised a method to enrich the informative regions of plasmids that had undergone sequential *in trans* and *in cis* recombination (Supplementary Fig. S4).

Briefly, the region containing the two cDNAs encoding interacting proteins were amplified by PCR using flanking oligonucleotides carrying PTO modifications at their 5′ end. The PCR products were then treated with a combination of DpnI, Lambda Exo, and Exonuclease I, to digest the plasmids and degrade single-stranded PCR products and linearized plasmids. This strategy resulted in a >50-fold enrichment of informative reads compared to direct sequencing of the plasmid pool (Supplementary Fig. S10A). Importantly, this enrichment did not substantially alter the representation of genes when compared to the non-enriched library (Supplementary Fig. S10B and C). Furthermore, PCR enrichment resulted in the loss of <10% of PPIs identified in the non-enriched samples, but this loss was more than compensated for by the large number of PPIs identified exclusively in the samples that had undergone PCR enrichment (Supplementary Fig. S10D and E). Details on the analysis of the data obtained from parallelized cDNA library screens can be found in the “Materials and methods” section, Fig. 2A and B, and Supplementary Figs S5 and S6.

**Figure 5.**
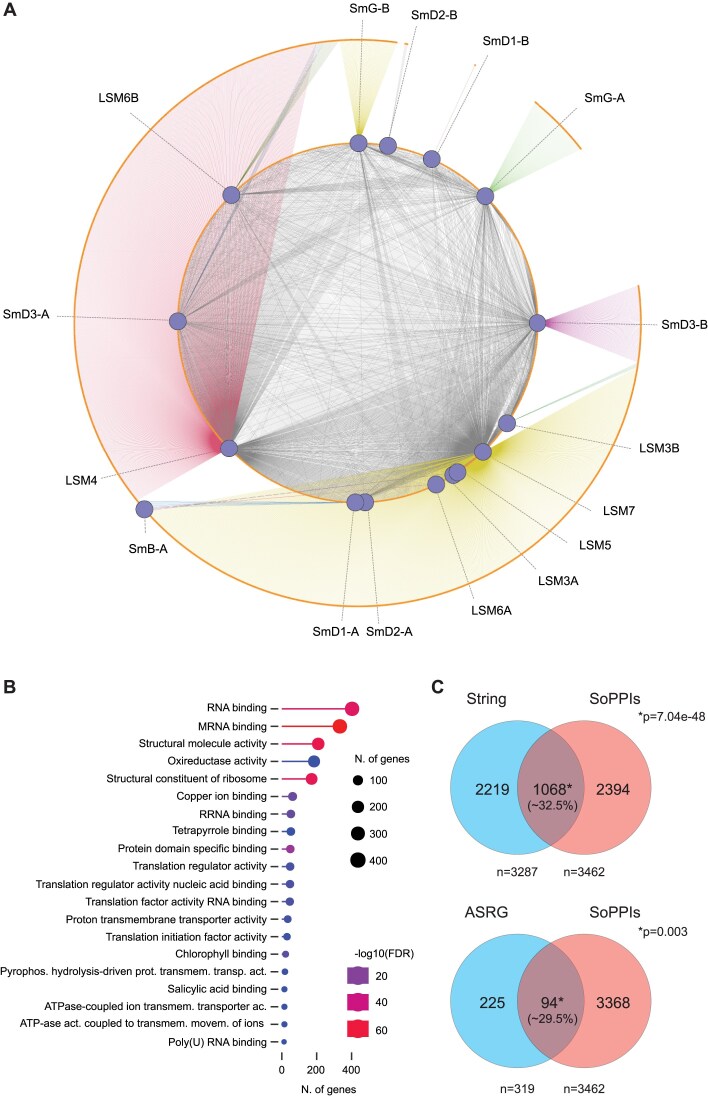
LSm/Sm PPI network generated using SoPPIs in *E. coli*. (**A**) Network of the PPIs with MS ≥1 (3213 interactions involving 2034 proteins) identified in parallelized SoPPIs cDNA library screens using 25 LSm/Sm proteins as bait. Colored lines indicate interactions specific to individual LSm/Sm proteins; gray lines connect interactors shared between two or more LSm/Sm proteins. (**B**) GO molecular function analysis of 2034 interactors with MS ≥1. (**C**) Venn diagram showing the overlap of 3462 proteins interacting with Arabidopsis LSm/Sm proteins identified using SoPPIs with String and ASRG databases. p-values are calculated on the cumulative distribution function of the hypergeometric distribution.

In total, parallelized cDNA library screens identified 11 018 PPIs involving 3462 proteins (Supplementary Fig. S11A and Supplementary Dataset S1). Applying a more stringent cut-off of MS ≥1 and considering only PPIs found in both biological replicates, we identified 3213 interactions involving 2034 Arabidopsis proteins (Fig. 5A and Supplementary Dataset S3). Among these proteins, Gene Ontology (GO) categories [76] related to RNA binding and mRNA binding, structural constituent of ribosome, rRNA binding, translation regulatory activity, translation regulator activity nucleic acid binding, translation factor activity RNA binding, translation initiation factor activity, and poly(U) RNA binding were highly enriched (Fig. 5B and Supplementary Fig. S11B and C), which is in agreement of the function of LSm/Sm proteins in mRNA maturation and RNA metabolism.

Similar results were obtained when only considering those 799 interactors originating from the cDNA library that were in frame with the N-Cre tag (Supplementary Fig. S11D), which might be overly stringent given that ribosomal frameshift slippage is known to occur in bacteria [77]. Other GO categories, such as those related to oxidoreductase activity, copper ion binding, chlorophyll-binding, and others, cannot intuitively be linked to RNA metabolism. These interactors could either indicate previously unknown functions of LSm/Sm proteins or constitute false-positive interactions. The latter seems possible as a non-normalized cDNA library was used, in which certain transcripts (e.g. genes involved in photosynthesis) are much more abundant than others (Supplementary Fig. S7D and E). Alternatively, these proteins may contain domains that facilitate interaction with LSm/Sm proteins that are not normally realized in a plant cell. However, the sheer number and significance of genes directly related to RNA metabolism confirm the overall quality of the data produced by SoPPIs. This notion is also supported by comparisons of our results obtained from screening the Arabidopsis cDNA library in duplicate with the String and Arabidopsis Splicing Related Genes (ASRG) (http://www.plantgdb.org/SRGD/ASRG/ASRP-home.php) databases [36–78]. In total, we identified 3 287 and 319 proteins in our cDNA library that are known or predicted to interact with LSm/Sm proteins, according to String and ASRG, respectively. Of these, 32.5% (1068) and 29.5% (94) were among the 3 462 LSm/Sm-interacting proteins identified in both SoPPIs replicates, representing a highly significant enrichment (Fig. 5C) and confirming the validity of our method.

## Discussion

Although the use of the split-Cre/loxP system to study PPIs and produce short DNA tags for sequencing has been described previously [10, 12], we have redesigned the system overcoming many of its earlier limitations.

In particular, the design of SoPPIs ensures that activation of a selectable marker occurs only after sequential *in trans* and *in cis* recombination. These recombinations are mediated by the reconstitution of functional Cre recombinase from two inactive fragments that associate upon interaction between two test proteins. In addition, SoPPIs plasmids include all elements required for the expression of the test proteins, as well as all selectable markers. As a result, specialized host strains such as *E. coli* strains expressing T7 polymerase or *S. cerevisiae* GAL4/ADE2 mutant strains are generally not required. Consequently, the method can be implemented in a wide variety of strains, offering both practical and economic advantages for the broader research community. Results obtained during the development of the method in *E. coli* clearly demonstrate that SoPPIs can detect PPI between soluble proteins with high specificity and reproducibility (Supplementary Fig. S3). However, like other 2-hybrid-based methods, SoPPIs in *E. coli* was unable to detect interactions between transmembrane proteins or interactions that require post-translational protein modifications. These limitations can be partially overcome by using a eukaryotic host such as *S. cerevisiae*, as demonstrated for SmB and SmD3 (Fig. 4E and Supplementary Fig. S8F), two proteins whose interaction requires methylation. Detection of PPIs that depend on other host-specific post-translation modifications may, however, required more specialized hosts. In this context it is important to note that SoPPIs could, in principle, be implemented in eukaryotic hosts other than yeast, provided that the cells can be transformed with and maintain episomal plasmids and that suitable selectable markers are available.

Another highly convenient feature of SoPPIs is that the entire procedure is performed in liquid culture, eliminating the need for plating and handling of individual colonies during or after selection using antibiotic or auxotrophic markers. This reduction in steps decreases both the space and time required for screening PPIs compared to current methods. Additionally, liquid cultures enable the recovery of a higher number of PPIs, thus increasing the depth and representativeness of the screen. Lastly, SoPPIs does not require expensive equipment or highly skilled operators. It has been designed to serve as a robust routine method for massively parallelizing PPI analyses, and it can be conducted in any laboratory equipped with standard molecular biology tools.

The substantial reductions in cost and time also make it feasible to perform PPI assays with multiple biological replicates, which has not been possible using standard techniques such as Y2H. This, in turn, reduces the number of false-positive PPI detected. Specifically, PPI assays using biological replicates will reduce the number of false positives arising from self-assembly of the two Cre fragments, which is a rare event to start with and thus extremely unlikely to occur consistently across multiple replicates. Furthermore, biological replicates will reduce the number of false-positive PPIs that might be caused by an occasional triple plasmid transformation, in which a third plasmid is randomly co-transformed along with two plasmids encoding a pair of genuinely interacting proteins. In such a case, the ORF encoding the third protein, which does not interact with either of the other two proteins, may still become incorporated into the recombined plasmid through the activity of Cre recombinase reconstituted by the genuine interaction of the other two proteins expressed in the cell. However, the probability of such an event occurring in multiple replicates is extremely low. Consistent with this notion, false positives were not a major problem in replicate SoPPIs experiments and we detected very few PPIs among the Arabidopsis LSm/Sm proteins not listed in the String database [36, 37] (Fig. 3C, Supplementary Fig. S9E, and Supplementary Dataset S2).

The ability to efficiently screen millions of PPIs simultaneously makes SoPPIs an ideal method for studying protein interactomes within organisms and between different organisms (e.g. host–pathogen interactions and hybrid phenotypic vigor in F1 generation of crops), as well as exploring new aspects of applied research. For instance, SoPPIs can in principle be employed to probe highly complex peptide libraries against one or multiple protein(s) of interest. The discovery of conserved peptides that specifically interact with a given protein of interest would open up entirely new avenues in both basic and applied research. These peptides could be utilized to target other domains of interest in specific tissues and cell compartments, as well as to direct markers (e.g. fluorophores and chemical-sensitive tags) for visualizing specific proteins both *in vivo* and *in vitro*. Additionally, SoPPIs can in theory be used to identify synthetic proteins (e.g. nanobodies) with specific properties, streamlining the process of producing such proteins in a reproducible, animal-free, and cost-effective manner.

Overall, our results establish SoPPIs as a powerful new technology to quickly and cost-effectively generate large PPI networks by screening millions of potential PPIs using multiple baits in parallel. SoPPIs overcomes many of the limitations normally associated with this type of experiment, i.e. the need for handling individual colonies and host-specificity. With its potential applications in both basic and applied research, SoPPIs represents a significant advancement in the field of proteomics.

## Supplementary Material

gkag716_Supplemental_Files

## Data Availability

Sequencing reads generated as part of this study have been deposited with the European Nucleotide Archive (ENA, https://www.ebi.ac.uk/ena/browser/), accession number PRJEB76943. The Python scripts used for the data analysis presented here are available as Jupyter Notebook files [32] through FigShare, accession number: https://doi.org/10.6084/m9.figshare.29236658.
