# Supplementary material for: SoPPIs: a highly parallelized protein–protein-interaction screening method in prokaryotic and eukaryotic hosts
Source: Nucleic Acids Res. 2026 Jul 22;54(14):gkag716. doi: 10.1093/nar/gkag716 (PMC13389306; doi:10.1093/nar/gkag716)
Supplement: gkag716_Supplemental_Files [file gkag716_supplemental_files.zip › Collani_et_al_Supplementary_R3-1.pdf]

Supplementary Information

SoPPIs: a highly parallelized protein-protein-interaction screening method in prokaryotic and eukaryotic hosts

Silvio Collani[1,2,*], Sarah Muniz Nardeli[1,3], K.V.S.K. Arjun Chowdary[3], Daniela Goretti[1,2], Markus Schmid[1,3,*]

[1]Umeå Plant Science Centre, Department of Plant Physiology, Umeå University, SE-901 87 Umeå, Sweden.
[2]DISSTE, University of Eastern Piedmont, 13100 Vercelli, Italy
[3]Department of Plant Biology, Linnean Center for Plant Biology, Swedish University of Agricultural Sciences, S-75007 Uppsala, Sweden.

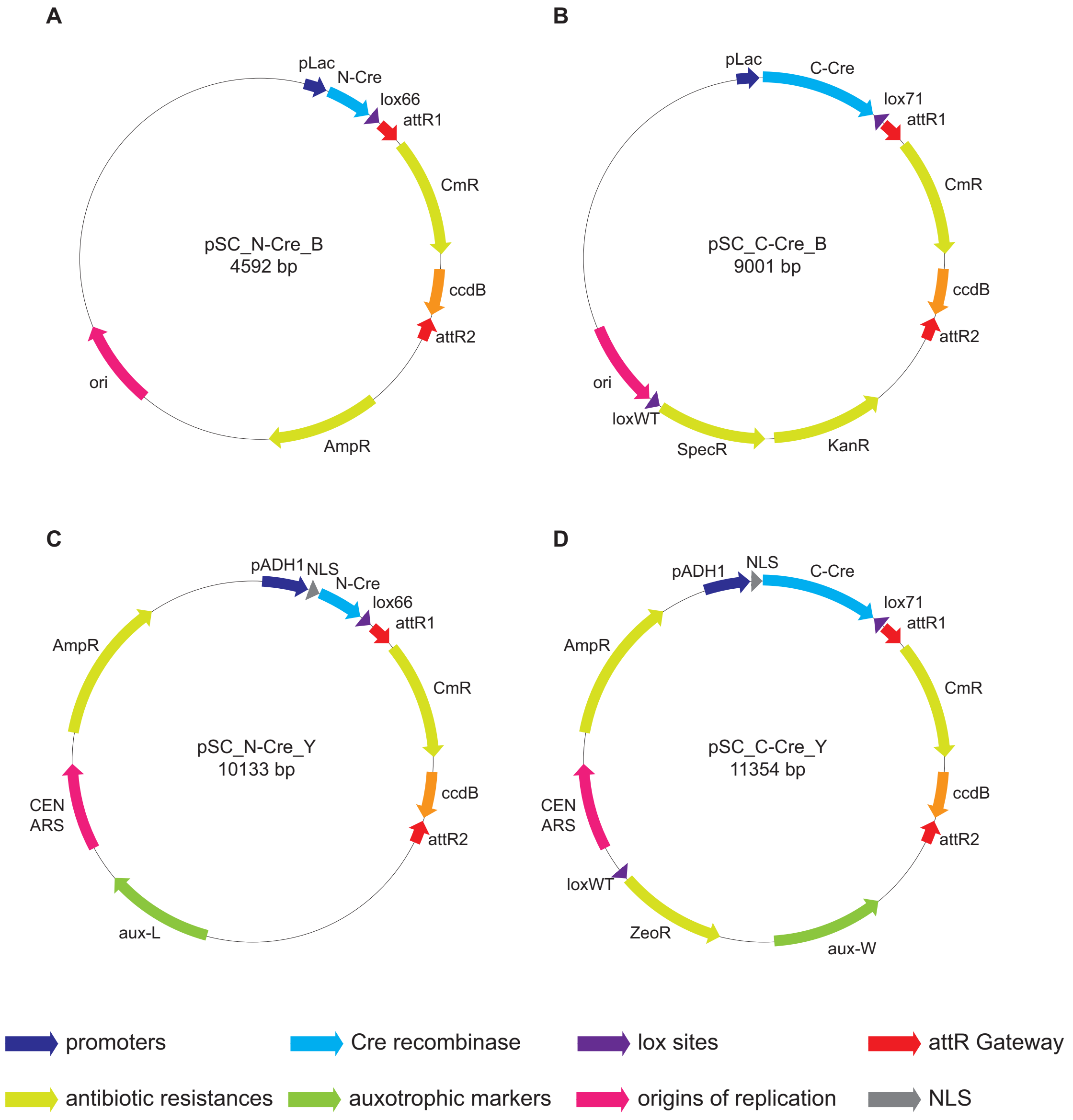

Supplementary Figure 1. Maps of the SoPPIs vectors. A) Vector for SoPPIs in bacteria providing the N-terminal fragment of the Cre recombinase, followed by a lox66 site, and the ccdB/chloramphenicol cassette flanked by attR Gateway recombination sites. The vector carries the constitutive resistance against ampicillin/carbenicillin. **B)** Vector for SoPPIs in bacteria providing the C-terminal fragment of the Cre recombinase, followed by a lox71 site, and the ccdB/chloramphenicol cassette flanked by attR Gateway recombination sites. The bacterial vectors carry genes conferring constitutive resistance against ampicillin/carbenicillin **(A)** and kanamycin **(B)** and a gene lacking a promoter and start codon providing resistance to spectinomycin **(B)** following sequential *in trans* and *in cis* recombination. **C)** Vector for SoPPIs in yeast providing the N-terminal fragment of the Cre recombinase, followed by a lox66 site, and the ccdB/chloramphenicol cassette flanked by attR Gateway recombination sites. The vector encodes a gene providing resistance against ampicillin/carbenicillin for selection in *E. coli* and the constitutive gene for leucine biosynthesis for the selection of yeast on a leucine-deficient medium. **D)** Vector for SoPPIs in yeast providing the C-terminal fragment of the Cre

recombinase, followed by a lox71 site, and the ccdB/chloramphenicol cassette flanked by attR Gateway recombination sites. The vector encodes a gene providing resistance against ampicillin/carbenicillin for selection in *E. coli* and the constitutive gene for tryptophan biosynthesis for selection in yeast, and an ORF lacking a start codon providing resistance to zeocin following sequential *in trans* and *in cis* recombination.

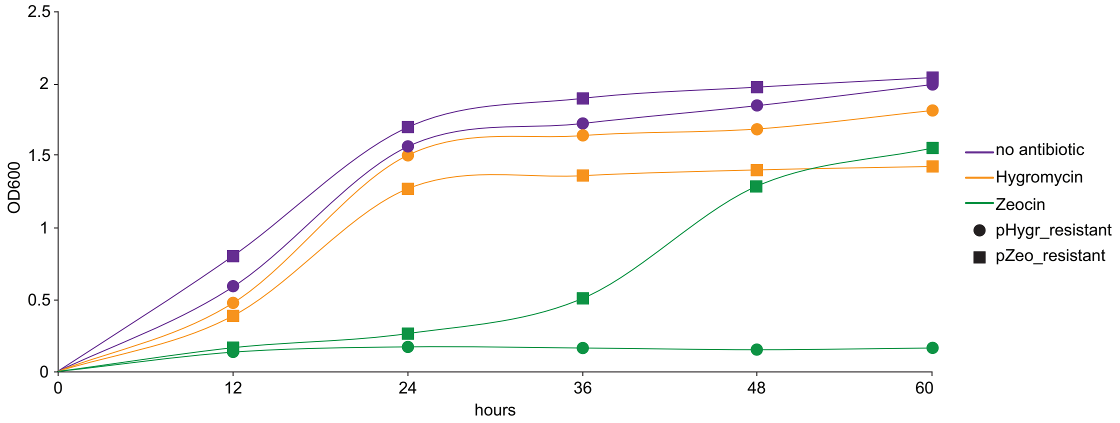

Supplementary Figure 2. Comparison of resistance to hygromycin and zeocin as selectable markers in yeast liquid cultures. To identify a suitable marker for the selection of yeast in liquid medium, two different vectors were created: one providing resistance agaist hygromycin and the other providing resistance against zeocin. Both resistance genes were expressed under the control of the constitutive promoter *pADH1*. Yeast strain AH109 was transformed with the two vectors and cultured at 30ºC in liquid media lacking (control) or supplemented with one or the other antibiotic. Yeast growth was monitored by measuring OD_{600} over time. Zeocin antibiotic gives better selection in liquid culture and was therefore adopted in the SoPPIs as a selectable marker in yeast.

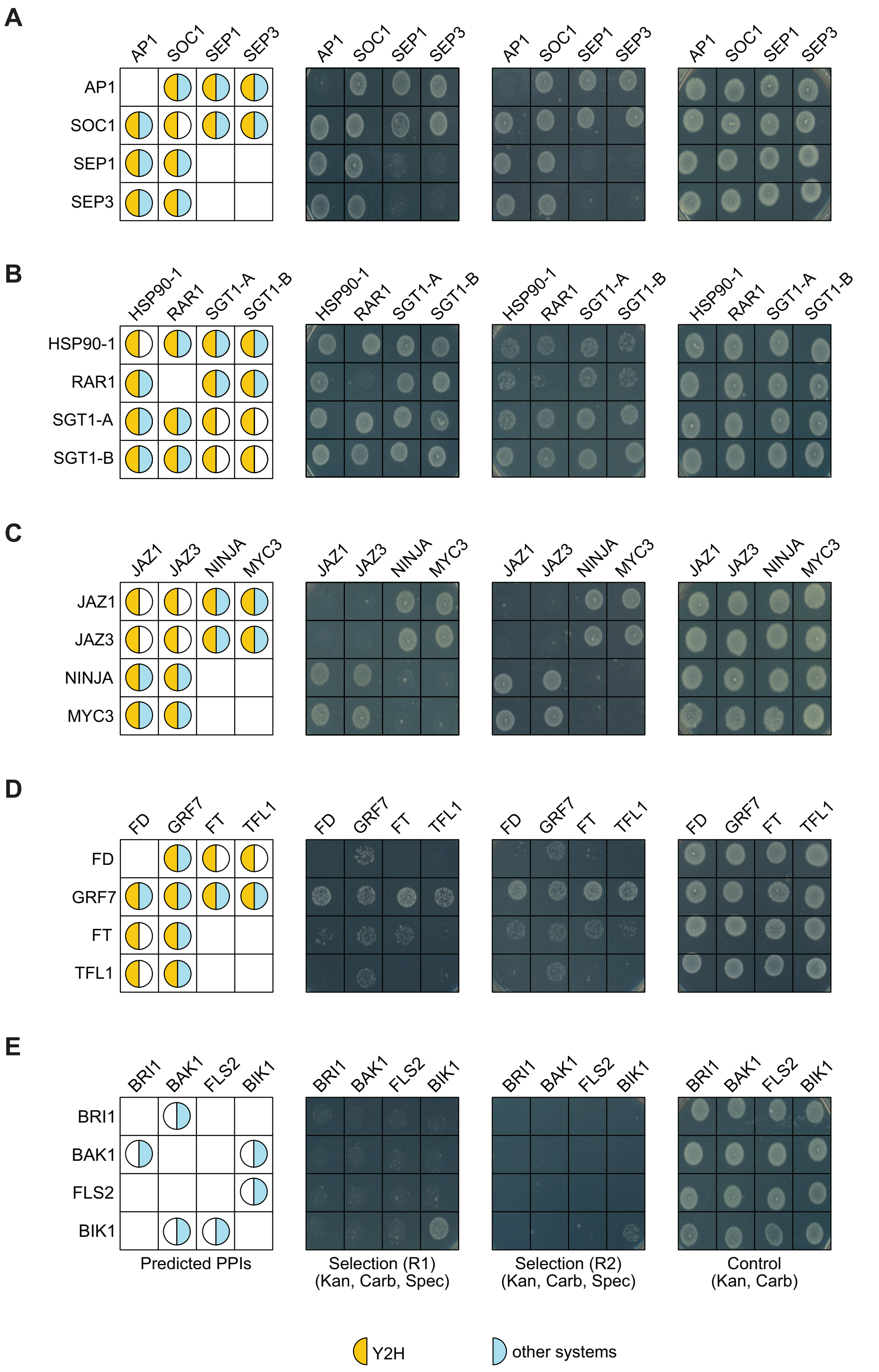

Supplementary Figure 3. Validation of SoPPIs in E. coli using Arabidopsis protein quartets. Each row (**A–E**) shows all 16 possible pairwise combinations among four proteins arranged in a 4×4 interaction matrix. The leftmost panel in each row summarizes protein-protein

interactions (PPIs) reported in the literature, including interactions previously demonstrated by yeast two-hybrid (Y2H; yellow semicircles) or by other methods (blue semicircles). The second and third panels show two independent biological replicates of the PPI matrix grown on triple-antibiotic selective medium. The rightmost panel shows the cotransformation control, in which cells were co-transformed with both N-Cre and C-Cre and grown on double-antibiotic selective medium. **A**) Quartet: AP1, SOC1, SEP1, and SEP3. **B**) Quartet: HSP90-1, RAR1, SGT1A, and SGT1B. **C**) Quartet: JAZ1, JAZ3, NINJA, and MYC3. **D**) Quartet: FD, GRF7, FT, and TFL1. **E**) Quartet: BRI1, BAK1, FLS2, and BIK1.

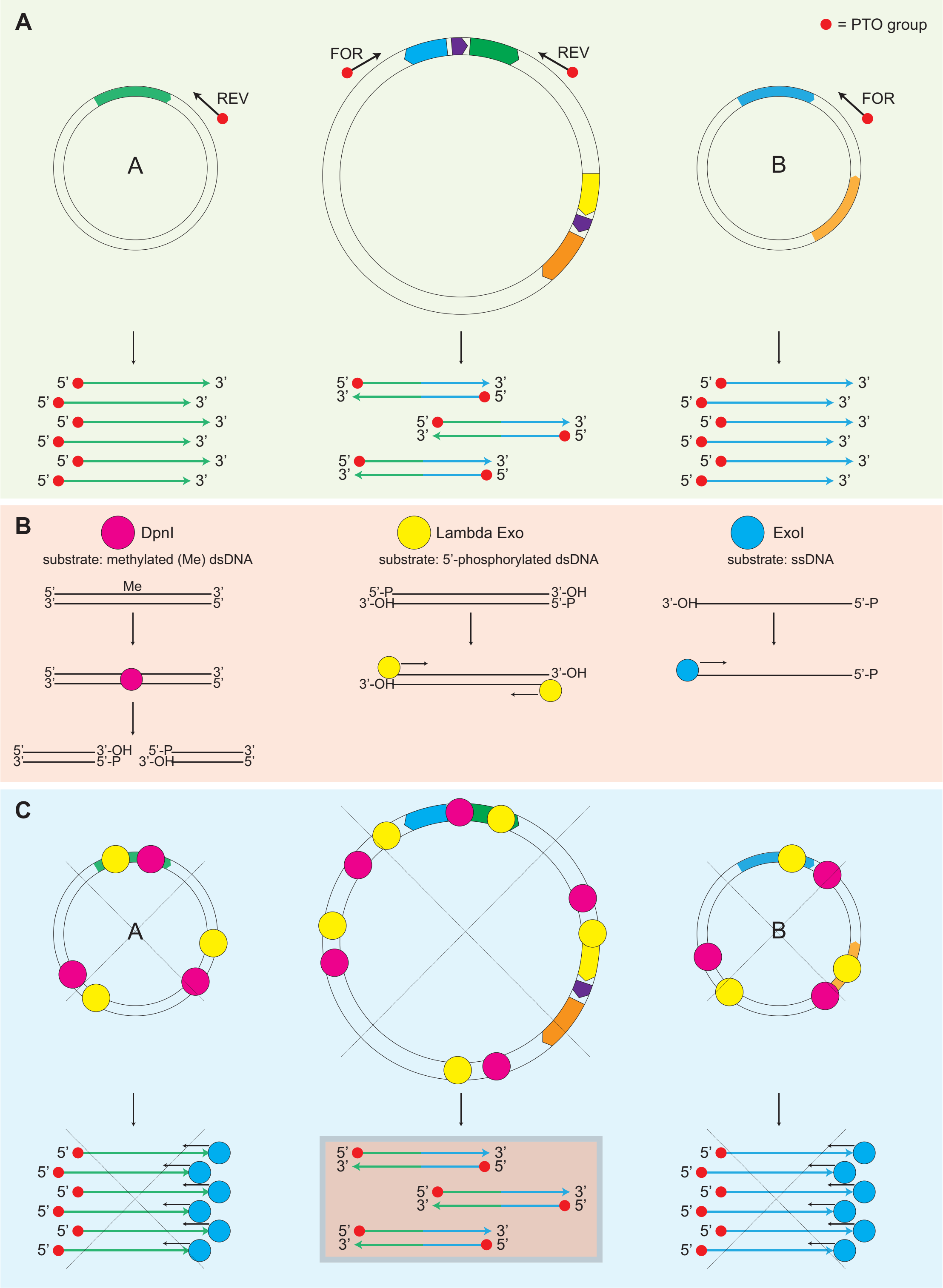

Supplementary Figure 4. Enrichment for sequential-recombined DNA fragments. A) After the selection phase, cells can contain three different plasmids: sequential recombined plasmids that have undergone *in trans* and *in cis* recombination, and the two original N-Cre and C-Cre plasmids. The original non-recombined plasmids are still present for several reasons: *i)* stoichiometry between the N-Cre and C-Cre plasmids is uneven, *i.e.* different from 1:1; *ii)* SoPPIs uses vectors with high-copy number origin of replication, resulting in a high and variable number of plasmids in the cell; *iii)* not all the recombinations occur at the same time. The DNA fragments originating from sequential recombined plasmids are enriched using a PCR-based strategy.

First, the sequential recombined region is amplified using two oligos (FOR; REV) flanking the sequences encoding the interacting proteins. Oligos are modified with a phosphorothioate group (PTO, red dot), which confers resistance against nucleases. During the PCR two types of linear DNAs will be produced: dsDNAs from the sequential recombined plasmids and ssDNAs from the non-recombined plasmids. **B)** Three different enzymes are used to degrade the DNA templates (*i.e.* recombined and non-recombined plasmids) and ssDNA PCR products. Plasmids are linearized using DpnI (pink circles), which recognizes methylated DNA (GA^{m6} | TC) sites generated by the *E. coli* strains used in SoPPIs (DH5α; DH10B). Linear DNA fragments not protected on both ends by PTO groups, *i.e.* DNA originating from the DpnI-digested plasmids and ssDNAs produced by PCR are then digested using a combination of Lambda Exonuclease (yellow circles), which is a 5' → 3' exodeoxyribonuclease that digests the 5'-phosphorylated strand of dsDNA and Exonuclease I (blue circles), which degrades ssDNA in 3' → 5' direction. The three nucleases are active in the same reaction buffer. **C)** PCR amplification and subsequent nuclease treatment results in the removal of template plasmids and unwanted PCR products (ssDNA; not protected on both ends by PTO groups), resulting in enrichment of linear DNA fragments consisting of the cDNAs encoding the interacting proteins, flanking the hybrid lox66/71 site.

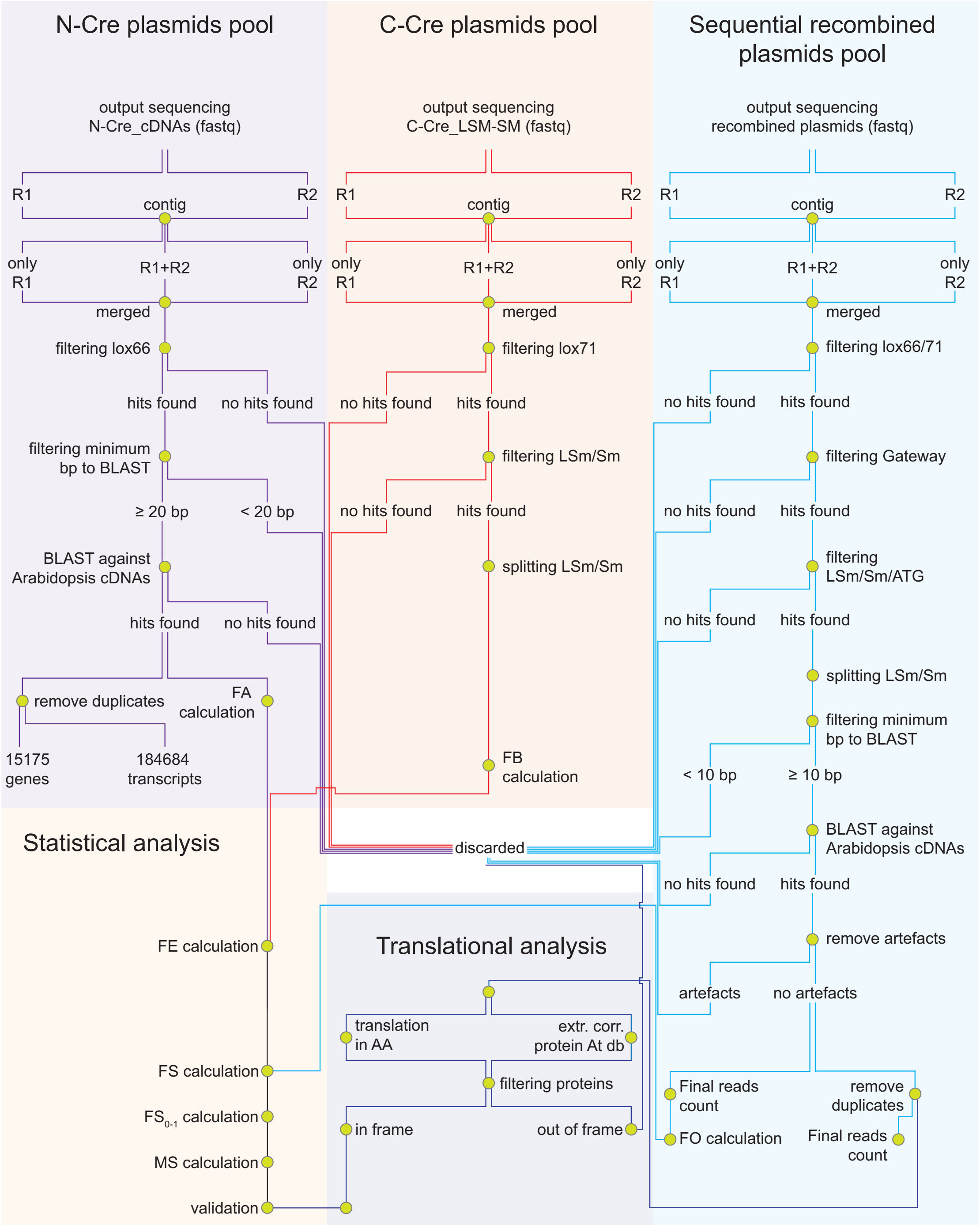

Supplementary Figure 5. Flowchart depicting the data analysis pipeline. The N-Cre plasmid pool (cDNA library), C-Cre plasmid pool (25 LSm/Sm genes), and sequential recombined plasmid pool were analyzed separately to calculate the frequencies FA, FB, and FO values, respectively. Statistical analyses were performed starting from FA and FB to calculate the frequency expected (FE) with which one would expect a particular PPI to occur. FE and FO were used to calculate the Frequency Ccore (FS) and the Median-normalized Score (MS) values. Detailed information regarding the data processing and statistical analysis are reported in the Materials and Methods section and Supplementary Figure 6.

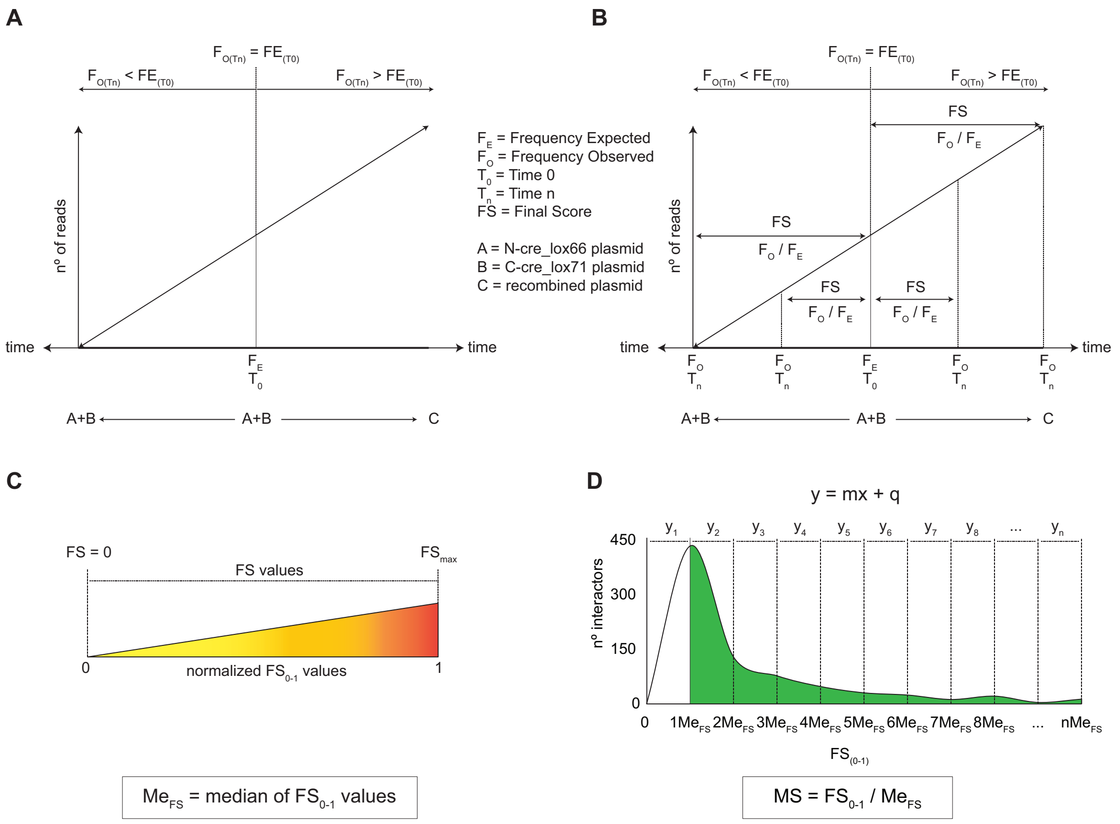

E

$$\text{FA (Frequence of reads A)} = \frac{\text{reads per gene}}{\text{total reads}}$$

$$\text{FB (Frequence of reads B)} = \frac{\text{reads per each LSm/Sm gene}}{\text{total LSm/Sm reads}}$$

$$\text{FE (Frequency Expected)} = \text{FA x FB}$$

$$\text{FO (Frequency Observed)} = \frac{\text{reads per each gene}}{\text{total reads}}$$

$$\text{FS (Frequency Score)} = \frac{FO}{FE}$$

$$FS_{0-1}\ \text{(FS normalized)} = \frac{FS}{FS_{max}}$$

$$Me_{FS} = \text{median of } FS_{0-1} \text{ values}$$

$$\text{MS (Median Score)} = \frac{FS_{0-1}}{Me_{FS}}$$

Supplementary Figure 6. Details of the statistical analysis. A) Cartesian plane showing the situation at the beginning of the experiment (T_0). At T_0, plasmids A and B are co-transformed into a cell. The Frequency Expected (FE) for each possible combination is calculated from the read numbers of the sequencing of the plasmid pools used for the co-transformation (FA x FB). In case of interaction among proteins encoded by plasmids A and B, their sequential recombination will produce plasmid C. At the end of the experiment (T_n) the Frequency Observed (FO) for each possible combination is calculated from the read numbers of the sequencing of the sequential recombined plasmids. FO can have values from 0 (no interaction at all) to any value where FO > FE. **B)** Cartesian plan showing the calculation of the Frequency Score (FS) for some hypothetical values of FO found at T_n. The FS is calculated as the ratio between FO and FE and can have values from 0 (no interaction) to infinite. **C)** Normalization of the FS values in a scale from 0 to 1 allowing comparison among different datasets of $FS_{(0-1)}$. FS values are normalized according to the maximum FS values FS_{max}. Normalized $FS_{(0-1)}$ values were then used to calculate the median value (Me_{FS}). **D)** Line graph showing

number of interactors in relation to the distribution of the $FS_{(0-1)}$ values grouped for their median (Me_{FS}) using multiples of Me_{FS} as x-axis unit. Each segment of the line is represented by an "$y_{(n-1)}$" and can be described by the linear equation y = mx + q, where m (angular coefficient; slope) corresponds to the difference of the values between the $Me_{FS(n)}$ and $Me_{FS(n-1)}$. Final MS values are calculated by the ratio of $FS_{(0-1)}$ to MS_{FS}. In principle, higher $FS_{(0-1)}$ values, and consequently higher MS values, indicated a higher likeliness for a particular PPI. Values in green represent all values with MS ≥ 1 and are the values considered significant in the experiment. **E)** Summary of the main formulas used in data analysis.

A

n° of reads Blasted	1023873
n° of genes	15175
n° of transcripts	184684

B

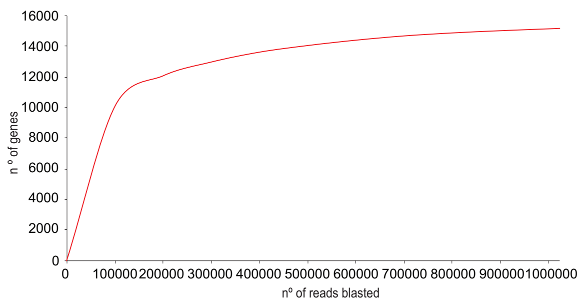

C

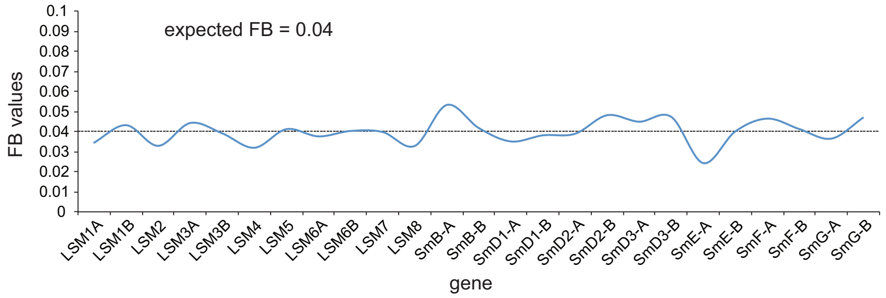

D

E

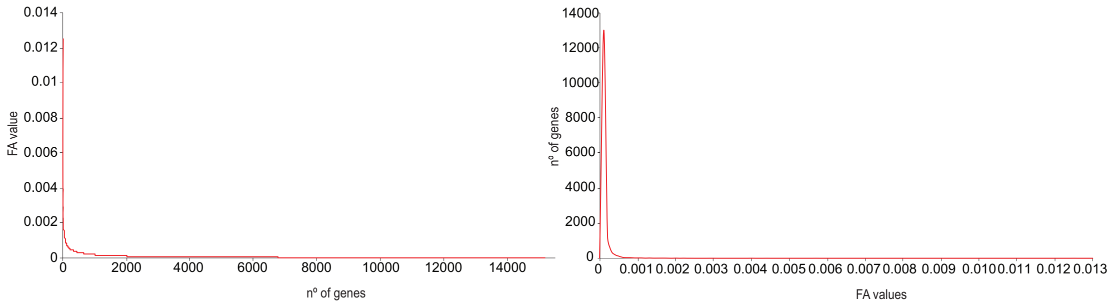

Supplementary Figure 7. Quality assessment of the N-Cre cDNA and the C-Cre (L)Sm plasmid pools. A) Number of sequences obtained by sequencing the Arabidopsis cDNA library in the SoPPIs N-Cre plasmid that resulted in a high-quality hit in a BlastN search against the Arabidopsis cDNA database. Number of genes and number of different transcripts (*i.e.*: different transcripts can encode for the same gene) identified (best hit) by the BlastN search. **B)** The 1023873 sequences that resulted in a BlastN hit (see panel A) is plotted against the number of genes uniquely identified in bins of 100 000 sequences. After approximately 700 000 sequences the number of new genes approaches a plateau, indicating that almost all the genes presented in the cDNA library were identified, *i.e.* confirm the complexity of the cDNA library at >15 000 genes. **C)** FB values for each of the 25 LSm/Sm genes after sequencing the pool of LSm/Sm C-Cre plasmids indicate a frequency of each gene at approximately 0.04 (4%), indicating that the 25 LSm/Sm genes are roughly equally represented in the plasmid pool. **D, E)** Frequency of genes present in the cDNA library. Genes are unevenly distributed, and a small number of transcripts has extremely high FA values. For example, only three genes encoding for members of the Rubisco small subunit (RBCS) multigene family count for almost 14% of the total reads (RBCS1A 8.8%, RBCS3B 3.8%, and RBCS2B 1.1%).

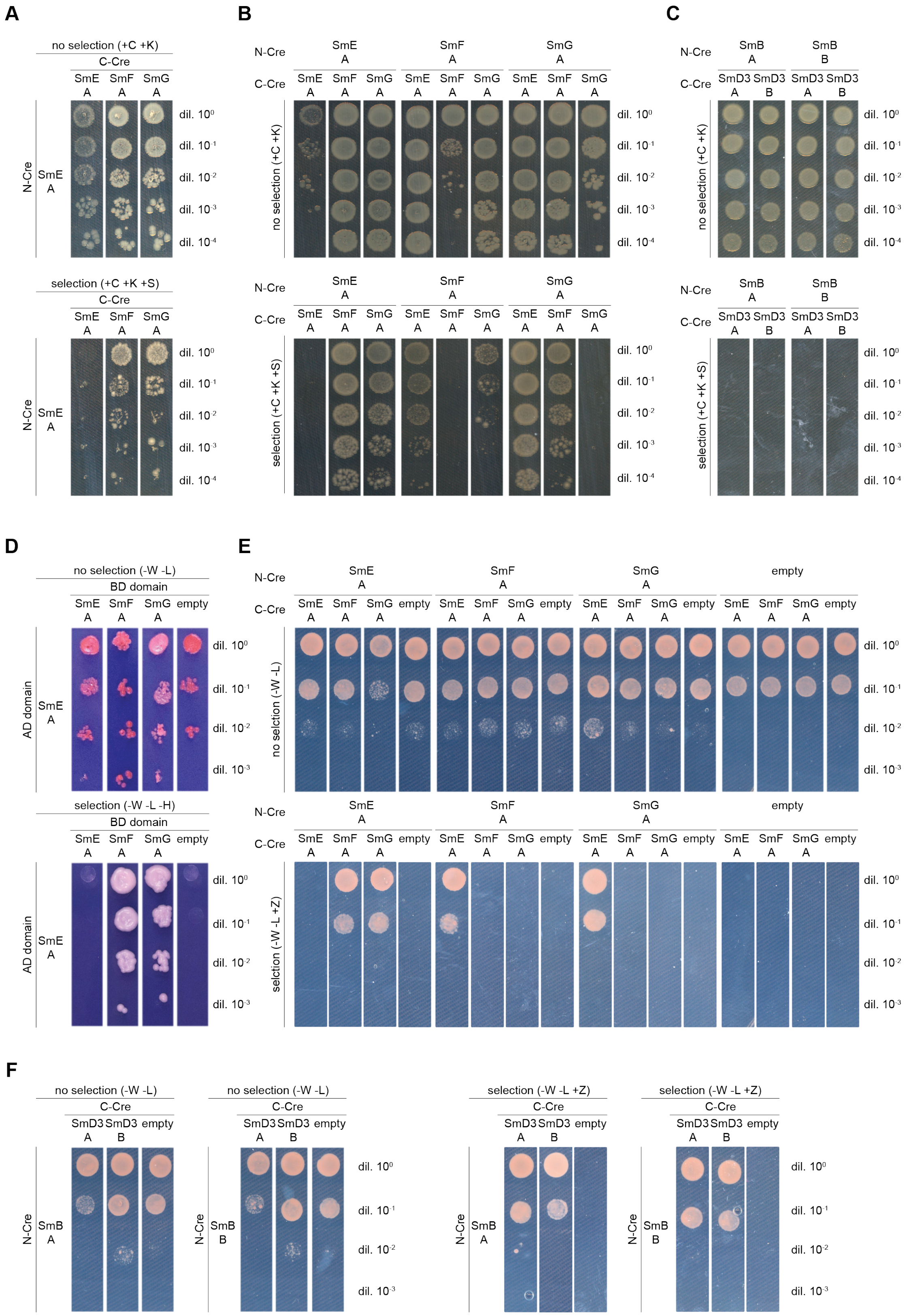

Supplementary Figure 8. Interaction of *Arabidopsis* Sm protein in *E. coli* and *S. cerevisiae* using SoPPIs. A) Serial dilutions of the experiment testing interaction between SmE-A and SmE-A, SmF-A, and SmG-A using SoPPIs in *E. coli* reported in Figure 2A. **B)** Serial dilutions of experiment testing interactions between SmE-A, SmF-A, and SmG-A using SoPPIs in *E. coli* reported in Figure 4B. **C)** Serial dilutions of the experiment testing interactions between SmB and SmD isoforms using SoPPIs in *E. coli* reported in Figure

4D. **D)** Serial dilutions of the experiment testing interaction between SmE-A and SmE-A, SmF-A, and SmG-A using Gal4-based Y2H in *S. cerevisiae* reported in Figure 2B. **E)** Serial dilutions of the experiment testing interactions between SmE-A, SmF-A, and SmG-A using SoPPIs in *S. cerevisiae* reported in Figure 4C. **F)** Serial dilutions of the experiment testing interactions between SmB and SmD isoforms using SoPPIs in *S. cerevisiae* reported in Figure 4E.

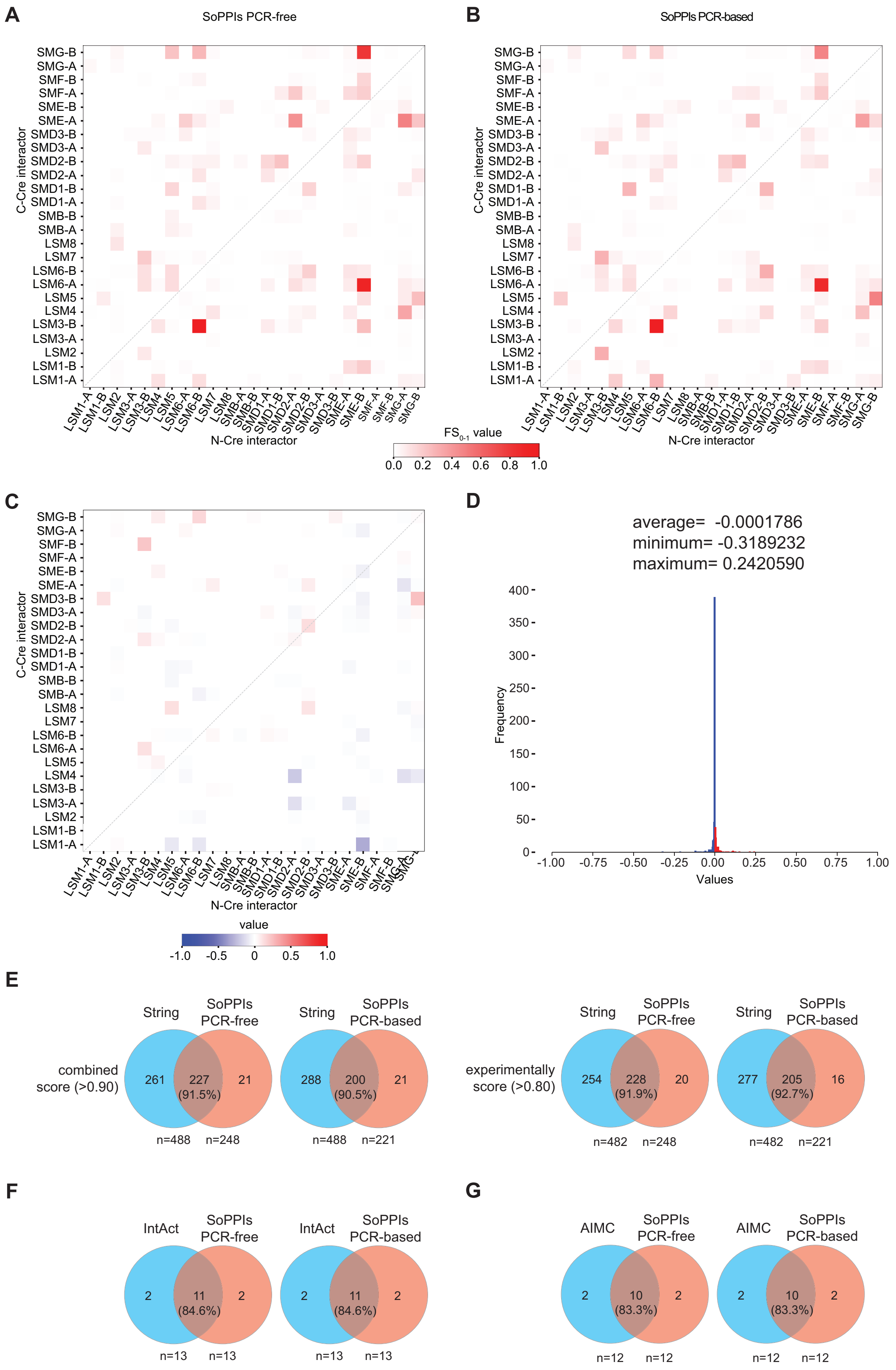

Supplementary Figure 9. Comparison of PCR-free and PCR-based NGS library preparation methods on the interaction matrix of 25x25 (L)Sm proteins. A, B) FS_{0-1} values for the full (25x25) matrix of *Arabidopsis* LSm/Sm proteins obtained using a PCR-free NGS library preparation method **(A)** and PCR-based method **(B)**. The FS_{0-1} score indicates the support for the

interaction, with 0 being very unlikely and values close to 1 indicating strong support that a particular interaction is real. **C)** Matrix with the differences of the FS_{0-1} values between PCR-free and PCR-based methods. Negative values indicate stronger support for a particular interaction using the PCR-based method whereas positive values indicate stronger support was observed using the PCR-free. Values around 0 indicate no significant difference in the FS_{0-1} values between the two methods. **D)** Distribution of the differences between FS_{0-1} values shown in C). The average of the differences between FS_{0-1}values is very close to 0, indicating that PCR-based and PCR-free NGS library preparation methods have only minor effects on SoPPIs results. **E)** Comparison of the results from SoPPIs using PCR-based and PCR-free NGS library preparation methods to interactions among LSm/Sm proteins predicted in the String database with two different filters applied: combined score > 0.90 and experimentally score > 0.80. **F)** Comparison of the results from SoPPIs using PCR-based and PCR-free NGS library preparation methods with LSm/Sm proteins predicted in IntAct database. **G)** Comparison of the results from SoPPIs using PCR-based and PCR-free NGS library preparation methods with LSm/Sm proteins predicted in the database from *Arabidopsis Interactive Mapping Consortium*.

A

		merged reads	total reads	informative reads	ratio (%)
no DNA-enr	biorep_1	36408193	62051502	26943	0.04
	biorep_2	25643309			
DNA-enr	biorep_1	40025128	87223080	1953061	2.24
	biorep_2	47197952			

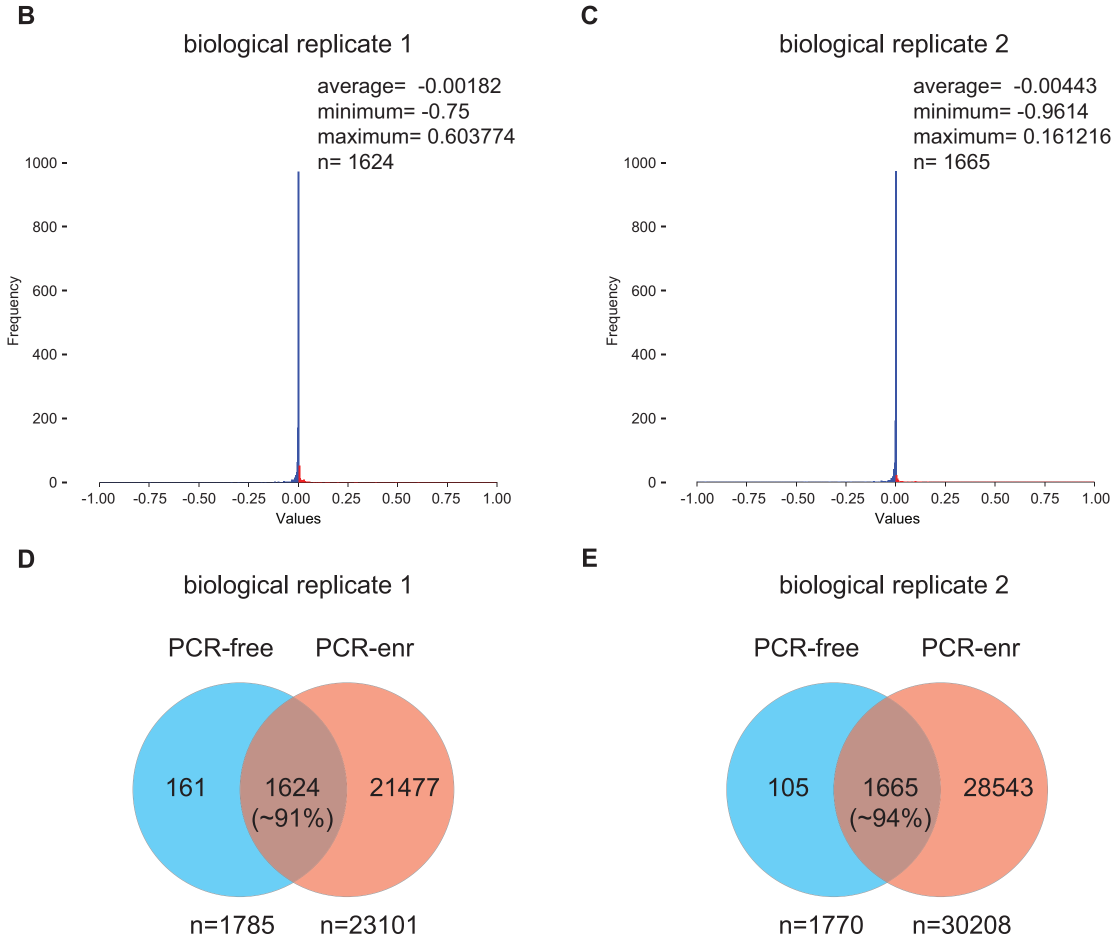

Supplementary Figure 10. Comparison of PCR-free and PCR-enriched NGS library preparation methods and results from parallelized cDNA library screens. A) Number of sequencing reads after contig building (merging paired-end reads, where possible) for two biological replicates using the non-enriched (top) and enriched (bottom) DNA for NGS library preparation. Informative reads are reads containing a lox66/71 site and sufficiently long flanking sequences to unambiguously identify the interacting proteins. Only 0.04% of the non-enriched sequencing reads were informative, whereas enrichment increased the proportion of informative reads to 2.24%, *i.e.* increases 56-fold. **B, C)** Difference of $FS_{0\text{-}1}$ values among the shared PPIs reported in biological replicates 1 **(B)** and 2 **(C)**. Values are extremely close to 0, demonstrating that enrichment for informative regions did not introduce any biases in PPI representation. **D, E)** Overlap of PPIs identified in the two biological replicates using non-enriched and enriched starting material for NGS library preparation. 91% and 94% of the PPIs are identified by both methods in replicates 1 **(D)** and 2 **(E)**, respectively, demonstrating their comparability.

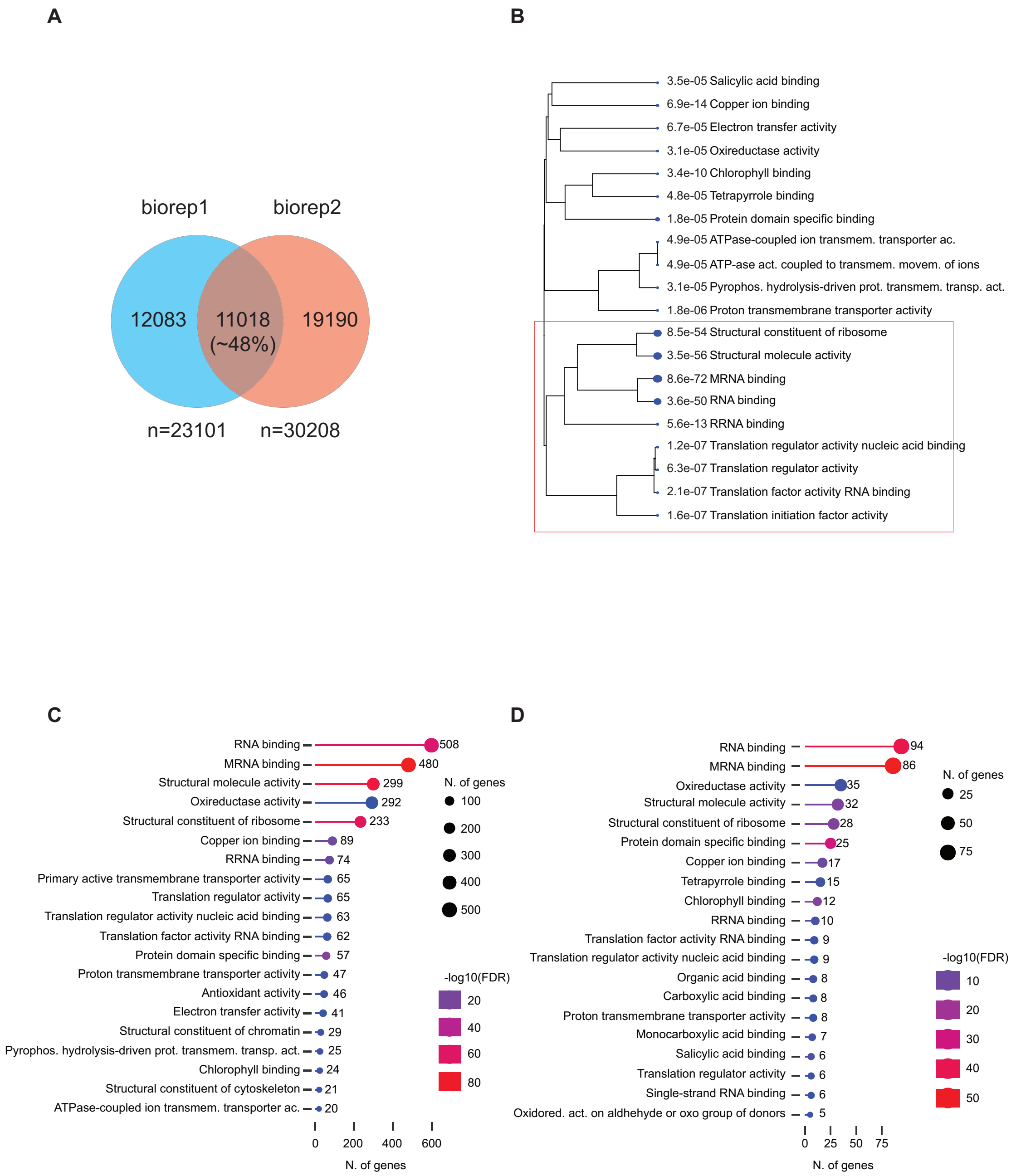

Supplementary Figure 11. PPIs between LSm/Sm and proteins encoded by cDNA library. A) A total of 11 018 interactions were shared between the two biological replicates (after enrichment for informative sequences) and considered for further analysis. **B)** Hierarchical clustering tree depicting the correlation among significant pathways after GO analysis. Pathways with many shared genes are clustered together. The size of the blue circle indicates the significance of the p-values. The red rectangle indicates the cluster enriched for GO categories directly related to RNA metabolism as expected for interactors with LSm/Sm proteins. **C)** GO molecular function analysis of all 3 462 interactors (irrespective of reading frame) with MS values higher than 0. **D)** GO molecular function analysis of those 799 interactors in frame with the N-Cre tag.

Supplementary Table S1. List of the genes used in the manuscript.

gene	accession ID
LSm1A	AT1G19120.1
LSm1B	AT3G14080.1
LSm2	AT1G03330.1
LSm3A	AT1G21190.1
LSm3B	AT1G76860.1
LSm4	AT5G27720.1
LSm5	AT5G48870.1
LSm6A	AT3G59810.1
LSm6B	AT2G43810.1
LSm7	AT2G03870.2
LSm8	AT1G65700.3
SmB-A	AT5G44500.1
SmB-B	AT4G20440.1
SmD1-A	AT3G07590.1
SmD1-B	AT4G02840.2
SmD2-A	AT2G47640.1
SmD2-B	AT3G62840.1
SmD3-A	AT1G76300.1
SmD3-B	AT1G20580.1
SmE-A	AT2G18740.1
SmE-B	AT4G30330.1
SmF-A	AT4G30220.2
SmF-B	AT2G14285.1
SmG-A	AT2G23930.1
SmG-B	AT3G11500.1
AP1	AT1G69120.1
SOC1	AT2G45660.1
SEP1	AT5G15800.1
SEP3	AT1G24260.2
HSP90-1	AT5G52640.1
RAR1	AT5G51700.1
SGT1-A	AT4G23570.1
SGT1-B	AT4G11260.1
JAZ1	AT1G19180.1
JAZ3	AT3G17860.1
NINJA	AT4G28910.1
MYC3	AT5G46760.1
FT	AT1G65480.1
FD	AT4G35900.1
TFL1	AT5G03840.1
GRF7	AT3G02520.2
BRI1	AT4G39400.1
BAK1	AT4G33430.1
FLS2	AT5G46330.1
BIK1	AT2G39660.1

Supplementary Table S2. List of the raw reads used in the manuscript and submitted to the European Nucleotide Archive (PRJEB76943).

method	experiment	library	replica	reads	seq. date	file name
iSeq 150 PE	25x25 PCR free	SoPPIs	NA	R1	04.03.22	S1_L001_R1_001.fastq.gz
				R2	04.03.22	S1_L001_R2_001.fastq.gz
				R1	04.03.22	S2_L001_R1_001.fastq.gz
				R2	04.03.22	S2_L001_R2_001.fastq.gz
				R1	04.03.22	S3_L001_R1_001.fastq.gz
				R2	04.03.22	S3_L001_R2_001.fastq.gz
		plasmids pool	NA	R1	04.03.22	S4_L001_R1_001.fastq.gz
				R2	04.03.22	S4_L001_R2_001.fastq.gz
	25x25 PCR based	SoPPIs	NA	R1	10.06.22	S1_L001_R1_001.fastq.gz
				R2	10.06.22	S1_L001_R2_001.fastq.gz
				R1	10.06.22	S2_L001_R1_001.fastq.gz
				R2	10.06.22	S2_L001_R2_001.fastq.gz
				R1	10.06.22	S3_L001_R1_001.fastq.gz
				R2	10.06.22	S3_L001_R2_001.fastq.gz
		plasmids pool	NA	R1	10.06.22	S4_L001_R1_001.fastq.gz
				R2	10.06.22	S4_L001_R2_001.fastq.gz
NovaSeq 150 PE	25xcDNAs no PCR-enrichment	SoPPIs	1	R1	12.06.23	B1_EKDL230008374-1A_HF5CYDSX7_L2_1.fq.gz
				R2	12.06.23	B1_EKDL230008374-1A_HF5CYDSX7_L2_2.fq.gz
			2	R1	12.06.23	B2_EKDL230008375-1A_HF5CYDSX7_L2_1.fq.gz
				R2	12.06.23	B2_EKDL230008375-1A_HF5CYDSX7_L2_2.fq.gz
	25xcDNAs with PCR-enrichment	SoPPIs	1	R1	21.10.23	B1_PCR_enr_EKDL230016604-1A_H7MGYDSX7_L2_1.fq.gz
				R2	21.10.23	B1_PCR_enr_EKDL230016604-1A_H7MGYDSX7_L2_2.fq.gz
				R1	19.11.23	B1_PCR_enr_EKDL230016604-1A_HVNLFDSX7_L1_1.fq.gz
				R2	19.11.23	B1_PCR_enr_EKDL230016604-1A_HVNLFDSX7_L1_2.fq.gz
			2	R1	21.10.23	B2_PCR_enr_EKDL230016605-1A_H7MGYDSX7_L2_1.fq.gz
				R2	21.10.23	B2_PCR_enr_EKDL230016605-1A_H7MGYDSX7_L2_2.fq.gz
				R1	19.11.23	B2_PCR_enr_EKDL230016605-1A_HVNLFDSX7_L1_1.fq.gz
				R2	19.11.23	B2_PCR_enr_EKDL230016605-1A_HVNLFDSX7_L1_2.fq.gz
	25xcDNAs initial pools	cDNAs pool	NA	R1	12.06.23	Ncre_pool_EKDL230008376-1A_HF5CYDSX7_L2_1.fq.gz
				R2	12.06.23	Ncre_pool_EKDL230008376-1A_HF5CYDSX7_L2_2.fq.gz
		cDNAs pool		R1	21.10.23	Ncre_pool_EKDL230016607-1A_H7MGYDSX7_L2_1.fq.gz
				R2	21.10.23	Ncre_pool_EKDL230016607-1A_H7MGYDSX7_L2_2.fq.gz
		cDNAs pool		R1	19.11.23	Ncre_pool_EKDL230016607-1A_HVNLFDSX7_L1_1.fq.gz
				R2	19.11.23	Ncre_pool_EKDL230016607-1A_HVNLFDSX7_L1_2.fq.gz
		plasmids pool	NA	R1	21.10.23	Ccre_pool_EKDL230016606-1A_H7MGYDSX7_L2_1.fq.gz
				R2	21.10.23	Ccre_pool_EKDL230016606-1A_H7MGYDSX7_L2_2.fq.gz
		plasmids pool		R1	19.11.23	Ccre_pool_EKDL230016606-1A_HVNLFDSX7_L1_1.fq.gz
				R2	19.11.23	Ccre_pool_EKDL230016606-1A_HVNLFDSX7_L1_2.fq.gz
